# Visible Spectral, CIELAB and Putative Chromophore-Class Screening of Plant and Agro-Food Residue Extracts

**DOI:** 10.3390/plants15172685

**Published:** 2026-09-01

**Authors:** Joan Masanet Martí, Antonio Belda-Antolí, Daniel López Rodríguez, Jorge Jordán-Núñez, Álvaro-Francisco Morote, Bàrbara Micó-Vicent

**Affiliations:** 1 Faculty of Sciences, University of Alicante, 03690 San Vicente del Raspeig, Alicante, Spain; 2 Department of Earth and Environmental Sciences, Faculty of Sciences, University of Alicante, 03690 San Vicente del Raspeig, Alicante, Spain; antonio.belda@ua.es; 3 Department of Applied Mathematics, Universitat Politècnica de València, Plaza Ferrándiz y Carbonell s/n, 03801 Alcoy, Alicante, Spain; dalorod@upv.es; 4 Department of Graphic Engineering, Universitat Politècnica de València, Plaza Ferrándiz y Carbonell s/n, 03801 Alcoy, Alicante, Spain; jorjornu@upv.es; 5 Department of Experimental and Social Sciences Education, Faculty of Teacher Training, University of Valencia, Av. dels Tarongers 4, 46022 Valencia, Valencia, Spain; alvaro.morote@uv.es

**Keywords:** plant pigments, natural colorants, agro-food residues, CIELAB, apparent absorbance, anthocyanins, betalains, carotenoids, melanoidins, ultrasound-assisted extraction

## Abstract

Replacing synthetic colorants with natural alternatives requires source-specific extraction strategies because pigment classes differ in polarity, pH response and thermal stability. This first-pass screening focused on six plant and agro-food matrices: red cabbage, *Iris germanica* petals, dragon fruit, orange peel, spent coffee grounds and pine bark. Each matrix was subjected to twelve extraction treatments combining water, ethanol, acetone 30%, acid/base modifiers, boiling water, freezing and ultrasound assistance. Extracts were evaluated by visible transmittance spectroscopy from 360 to 740 nm, operationally transformed to apparent optical attenuation according to Aapp (λ) = −log10 [%T (λ)/100] only where measured %T exceeded 0.01; this signal may include both absorption and light scattering, and converted to CIELAB coordinates under D65/10° conditions. The combined spectral and colorimetric approach separated the extracts into red-purple and yellow-brown groups. Red cabbage and *Iris* showed anthocyanin-like behaviour, dragon fruit showed a betalain-compatible magenta response, orange peel showed carotenoid-compatible short-wavelength attenuation, and coffee grounds and pine bark generated broad brown-yellow profiles. Pigment-family assignments remain putative because chromatographic identification was not performed. This study is explicitly intended as a proof-of-concept descriptive mapping step rather than as a statistically validated extraction optimisation. Because every matrix–treatment combination was prepared once, all comparisons in this article describe the observed screening preparations only; they do not estimate reproducible treatment effects, confidence intervals or statistical significance. The previous equal-weight low-L*/high-C*ab intensity score has been removed from the revised analysis. No extraction yield, recovery percentage, mass balance, purity, pigment concentration or application performance was measured; accordingly, this study describes the optical appearance of the obtained extracts and does not quantify colorant recovery. Visible transmittance, apparent optical attenuation and CIELAB coordinates support only literature-informed class-level hypotheses and cannot identify a pigment family or individual compound.

## 1. Introduction

Natural colorants are receiving renewed attention because food, packaging, textile and coating sectors are seeking lower-impact alternatives to selected synthetic colorants and dyes. The drivers differ by application: in food, consumer preference and regulatory scrutiny are central, whereas in textile or industrial dyeing the persistence of dye-containing effluents is a major environmental concern [1,2,3,4,5,6]. Plant pigments and pigment-rich residues therefore offer a dual opportunity: they can supply colour and contribute to circular bioeconomy strategies by valorising horticultural, agro-food and forestry by-products [2,6].

The practical value of a natural colorant is not defined only by its biological origin. It depends on the chemical class of the chromophore, its solubility, pH and thermal response, stability to oxygen and light, and compatibility with the final application matrix [1,2,6,7]. Anthocyanins, betalains and carotenoids are structurally distinct chromophore families. Anthocyanins are flavonoid pigments whose colour is controlled by flavylium, quinoidal, carbinol and chalcone equilibria; betalains are tyrosine-derived immonium conjugates built around betalamic acid; carotenoids are long polyene tetraterpenoids; and brown colourants from coffee, bark or fungi often arise from polymeric or oxidised mixtures rather than from a single narrow-band chromophore [7,8,9,10,11,12,13,14,15,16,17,18,19,20,21].

The matrices selected in this study were chosen to cover pigment classes and feedstock types relevant to plant pigment recovery. Red cabbage and *Iris germanica* petals were included as anthocyanin-like materials with strong pH sensitivity [8,16,17,22]. Dragon fruit was included as a betalain-compatible matrix because betacyanins produce intense red-purple colours and absorb in the green visible region around 535–540 nm [9,18,23,24]. Orange peel was selected as an abundant citrus by-product containing carotenoids, especially xanthophylls and apocarotenoid derivatives associated with yellow-orange colour [10,25,26]. Spent coffee grounds and pine bark were selected as residues rich in melanoidins, phenolic acids, tannins and proanthocyanidins [11,12,13,14,19,20]. The fungal *Pisolithus* comparator from the original campaign is not part of the main plant-focused analysis; it is retained only in the Supplementary Materials as a non-plant test of workflow generality [15,21]. These source-class associations are literature-derived prior hypotheses used to frame the optical observations; they are not analytical identifications of the compounds present in the measured extracts.

The objective was to perform a first-pass optical screening of the visible transmittance, apparent optical attenuation and CIELAB behaviour of these matrices after twelve extraction treatments. The working hypothesis was that each matrix would display a distinct optical response and that the measured optical appearance would vary by matrix and composite extraction scenario. This design is therefore presented as a proof-of-concept, first-pass descriptive screening exercise rather than as an optimisation study: its purpose is to document matrix–scenario observations that warrant replicated chemical characterisation and subsequent process development. This study does not measure colorant recovery as an outcome: no extraction yield, recovery percentage, mass balance, purity, pigment concentration or application performance was determined.

## 2. Results

### 2.1. Overall Chromatic and Spectral Differentiation

All matrices yielded coloured liquid extracts under at least one extraction condition, indicating the extraction of light-absorbing compounds. The complete a* and b* coordinates for every matrix–treatment combination are reported in Table 1. The visible appearance, CIELAB coordinates and spectra separated the materials into two broad chromatic groups: red-purple matrices (red cabbage, *Iris germanica* and dragon fruit) and yellow-orange/brown matrices (orange peel, spent coffee grounds and pine bark). Complete paired transmittance and apparent-optical-attenuation profiles for the six plant/agro-food matrices are shown in Supplementary Figures S1–S6; the non-plant *Pisolithus* comparator is reported separately in Supplementary Figure S7; the main-text spectral figures emphasize the comparisons discussed below. Accordingly, the descriptive comparisons reported below map the measured appearance of the specific preparations and do not establish optimised extraction conditions, treatment ranks or statistically validated superiority. Throughout the Results, reported extrema refer only to individual CIELAB coordinates observed in these single preparations and must not be interpreted as replicated evidence that one treatment caused superior extraction, pigment recovery or application performance. No individual process-variable effect is inferred from these composite scenarios. Because the starting masses were not normalised to dry matter, all cross-matrix plots and tables are maps of observed colour appearance under the as-received-mass protocol; they are not comparisons of extraction yield, recovery efficiency or pigment abundance. In addition, dragon fruit is shown descriptively but is not assigned a valid treatment ranking because its filtration/dilution history was not standardised or recoverable.

**Figure 1:**
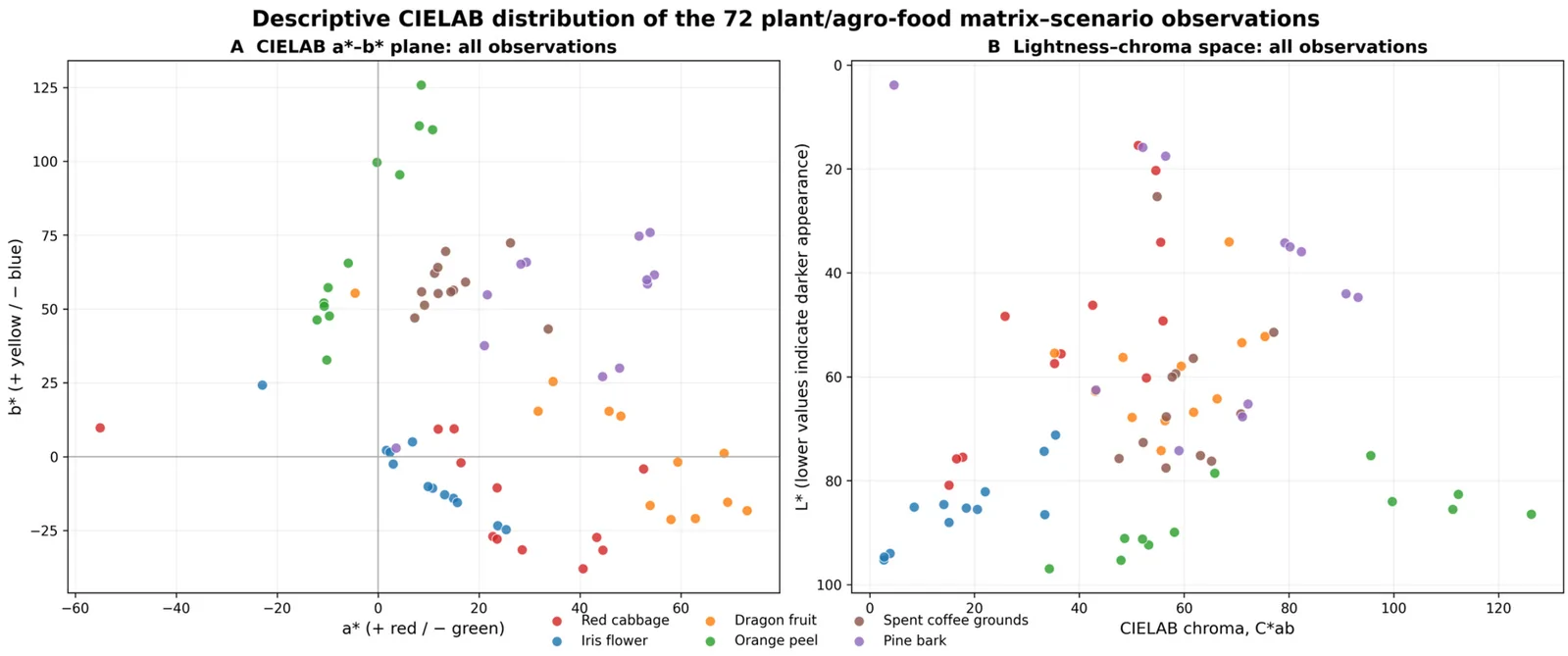
Descriptive CIELAB distribution of all 72 plant/agro-food matrix–scenario observations. (**A**) All observations in the CIELAB a*-b* plane. (**B**) All observations in lightness–chroma space, with the L* axis inverted so darker appearances plot higher. No composite score or treatment ranking is applied. The coordinates describe measured sample appearance, may be influenced by turbidity and scattering, and do not represent dry-matter-normalised pigment recovery.

**Figure 2:**
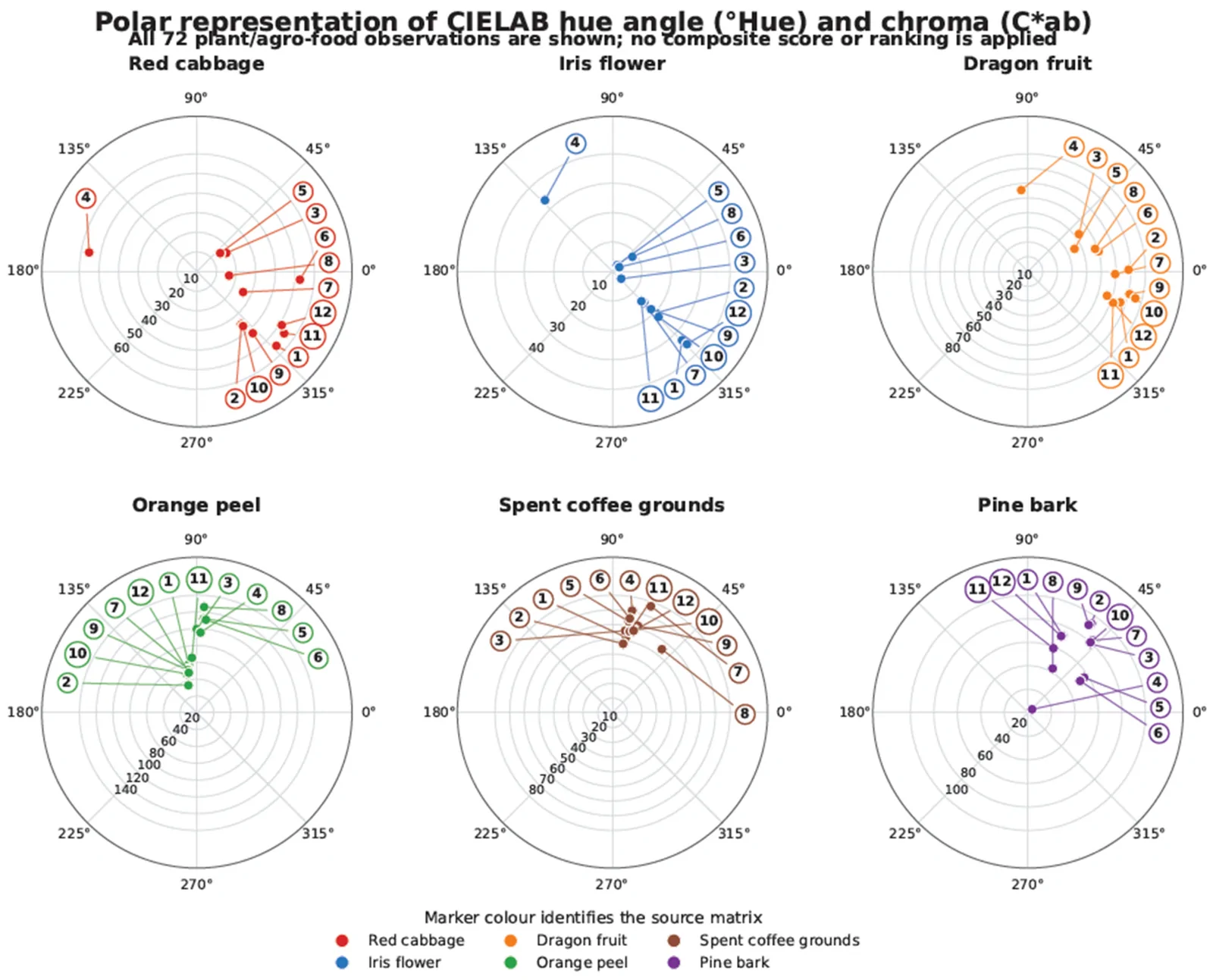
Polar representation of CIELAB hue angle (°Hue) versus chroma (C*ab) for all 72 plant/agro-food matrix–scenario observations. Angular position represents °Hue and radial distance represents C*ab. Treatment numbers are displayed in an external label ring and connected to their corresponding observations by leader lines to prevent overlap. Marker colours identify the source matrix: red, red cabbage; blue, Iris flower; orange, dragon fruit; green, orange peel; brown, spent coffee grounds; and purple, pine bark. No observation is selected or ranked. Dragon fruit observations are retained for descriptive colour-space completeness but are not quantitatively compared because the preprocessing details could not be recovered.

The CIELAB distribution supported this grouping (Figure 1A). Red cabbage, *Iris* and dragon fruit occupied regions with positive a* and negative or near-zero b*, consistent with red-purple optical behaviour. Orange peel, spent coffee grounds, and pine bark occupied regions with positive b*, consistent with yellow-brown optical behaviour. The L*-C*ab representation (Figure 1B) shows that the darkest and most chromatic observations were not always the same scenario. These coordinates are therefore reported independently, without combining them into a composite score. Because CIELAB colour position is jointly described by hue angle and chroma, Figure 2 additionally represents the complete treatment dataset in polar coordinates, using °Hue as the angular coordinate and C*ab as the radial coordinate. The independent within-matrix CIELAB extrema are summarized in Table 2.

The revised table retains °Hue because hue direction is an independent component of CIELAB colour description, but L*, C*ab and °Hue are not combined into a single index. Reporting separate extrema is transparent and reproducible, whereas an equal-weight composite score would impose an unvalidated preference for darkness and chroma and could be confounded by turbidity, oxidation or degradation. The extrema therefore describe the measured dataset only and do not constitute treatment selection or validation.

**Figure 3:**
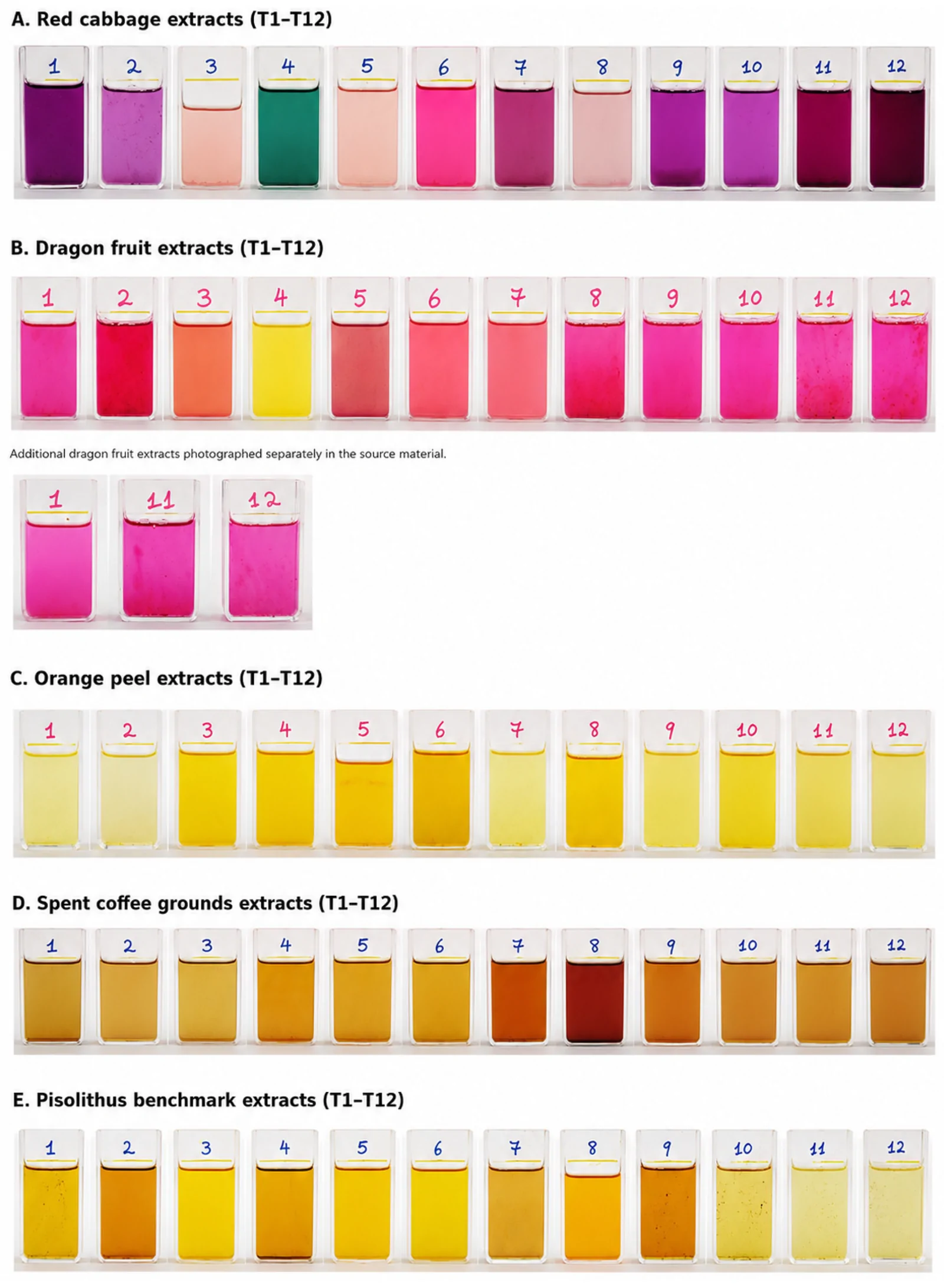
Photographic appearance of representative liquid extracts from the source screening record. Panels show (**A**) red cabbage, (**B**) dragon fruit, (**C**) orange peel and (**D**) spent coffee grounds. Handwritten numbers correspond to extraction treatments T1–T12. The dragon fruit photographs originated from two separate source images: the main row displays treatments T1–T12 at a matched scale, while additional photographs of T1, T11 and T12 are shown below for documentary completeness. The photographs provide a qualitative record of visible hue and intensity differences and should be interpreted together with the CIELAB coordinates and spectral data rather than as quantitative colour measurements.

Representative photographs of the extracts were retained as a qualitative visual record (Figure 3). They are not used as quantitative evidence, but they document hue changes that complement the calculated CIELAB coordinates and apparent-optical-attenuation profiles, including the green-blue shift in the KOH-added red-cabbage scenario, the magenta/yellow contrast in dragon fruit, the yellow-orange citrus extracts and the broad brown-yellow residue profiles. For the dragon fruit set, the individual treatment photographs have been reformatted to a standardized matched-size layout to facilitate direct visual comparison across treatments, and treatment T8 is now explicitly indicated because it was not present in the original composite source image.

### 2.2. Red-Purple Matrices: Red Cabbage, Iris Petals and Dragon Fruit

Positive transmittance values were converted to apparent optical attenuation to facilitate inspection of spectral shape; however, Aapp may combine molecular absorption with light scattering from suspended particles. Within the red-cabbage records, T12 had the lowest observed L* (15.46), whereas T4 had the highest observed C*ab (55.92). These independent extrema do not define a best treatment, extraction yield or colorant recovery. T11 gave L* = 20.30, a* = 44.55, b* = −31.57 and C*ab = 54.60 and displayed a green-region attenuation feature that is optically compatible with the broad response reported in the literature for anthocyanin-containing red cabbage [8,16,17]. The literature identifies cyanidin-derived anthocyanins in red cabbage, but the present measurements do not establish that any cyanidin glycoside, other anthocyanin or individual compound was present in the measured extract. The interpretation is therefore limited to an anthocyanin-like class-level hypothesis, and no quantitative comparison is made because turbidity was not measured and the optical profiles were not validated against conventional UV–visible spectrophotometry.

**Figure 4:**
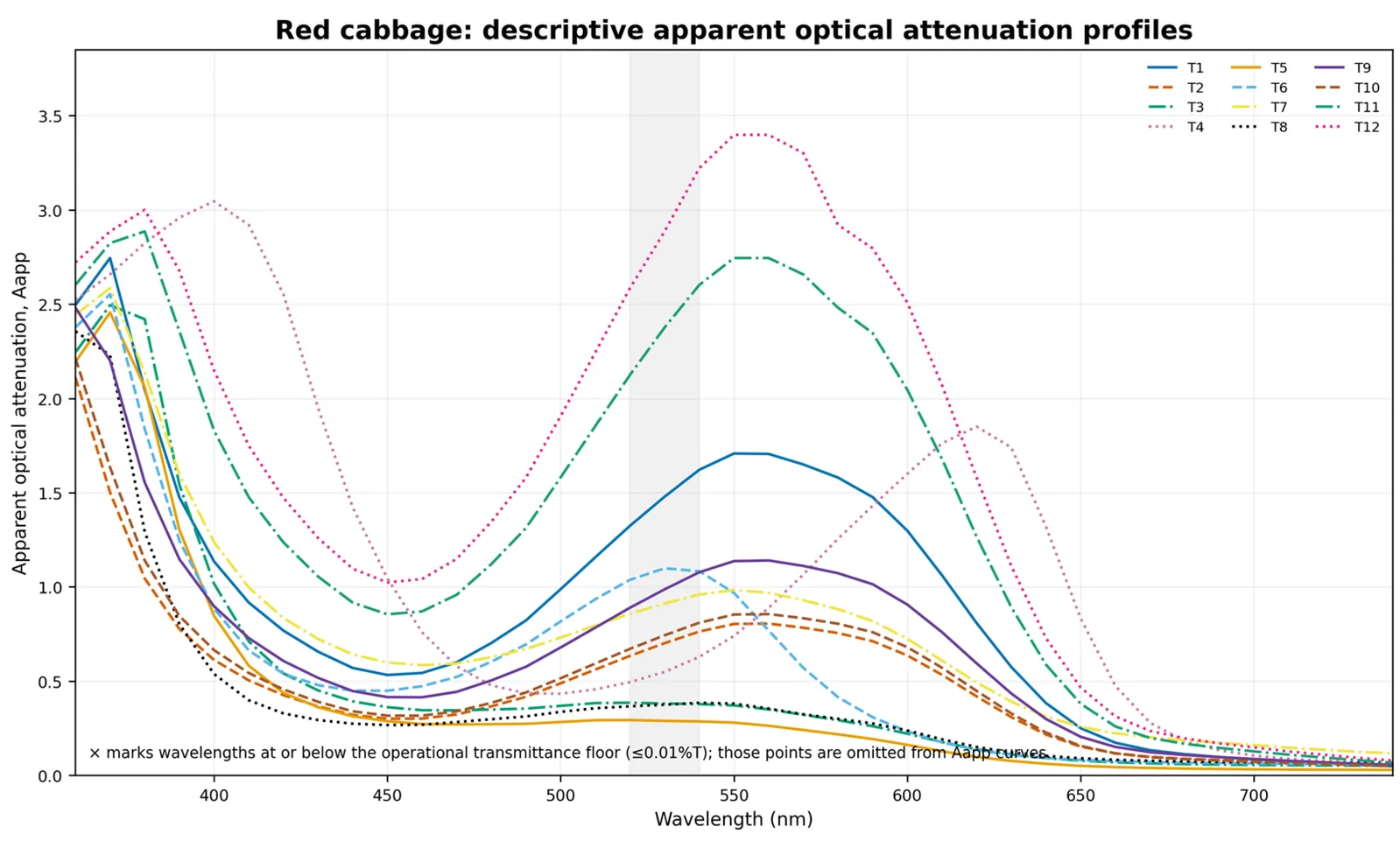
Descriptive apparent-optical-attenuation profiles of all twelve red-cabbage scenarios derived from measured transmittance. Aapp is shown only where %T > 0.01. × markers denote wavelengths at or below the operational transmittance floor; those points are censored and omitted from the curves. The shaded region indicates 520–540 nm for qualitative comparison only. No curve is designated as highest-ranked or quantitatively superior.

The KOH-added scenario T4 showed a different optical pattern from the other red-cabbage scenarios. In this single preparation, T4 had a high observed C*ab value, while its dominant response shifted toward longer wavelengths, with a maximum near the red region and an associated green-blue visual change; this descriptive observation is not evidence of superior treatment performance. This optical behaviour is compatible with a possible pH-related transformation of anthocyanin-like chromophores rather than simply indicating improved pigment recovery; however, no specific anthocyanin structure or conversion product is confirmed by the present measurements. In red-cabbage anthocyanin systems, increasing pH promotes transitions from the red flavylium cation toward quinoidal bases and, at higher pH, unstable chalcone-like species; this explains why alkalinity can create visually striking colours while simultaneously reducing relevance for stable food-grade red-purple colourant preservation [8,16]. Because final pH was not measured, the proposed pH mechanism remains a literature-informed hypothesis rather than a measured explanation of T4. The complete red-cabbage optical-attenuation profiles are presented in Figure 4.

*Iris germanica* records occupied a similar qualitative colour region; any apparent difference in lightness or chroma relative to red cabbage is descriptive only and does not indicate lower or higher treatment performance. In the Iris records, T12 had both the lowest observed L* (71.18) and the highest observed C*ab (35.42) among the twelve scenarios; this is a descriptive within-batch extremum, not a validated rank. The main apparent-optical-attenuation feature occurred in the same broad green-region window as red cabbage, supporting an anthocyanin-like interpretation. *Iris* petals are known to contain anthocyanin pigments and may also show colour shifts through metal complexation and co-pigmentation phenomena [22]. Because the sample masses were not normalised to dry matter, the lower C*ab and higher L* values observed for Iris relative to red cabbage describe only the appearance of the measured extracts and do not establish lower extraction efficiency or lower pigment abundance; the T12 observation was an extremum in the available CIELAB records; this single observation does not demonstrate that freezing plus ultrasound increased recovery, caused the response, or would be reproducible. Because turbidity was not quantified, even this optical extremum cannot be interpreted as pigment concentration. The anthocyanin-like class assignment remains putative.

**Figure 5:**
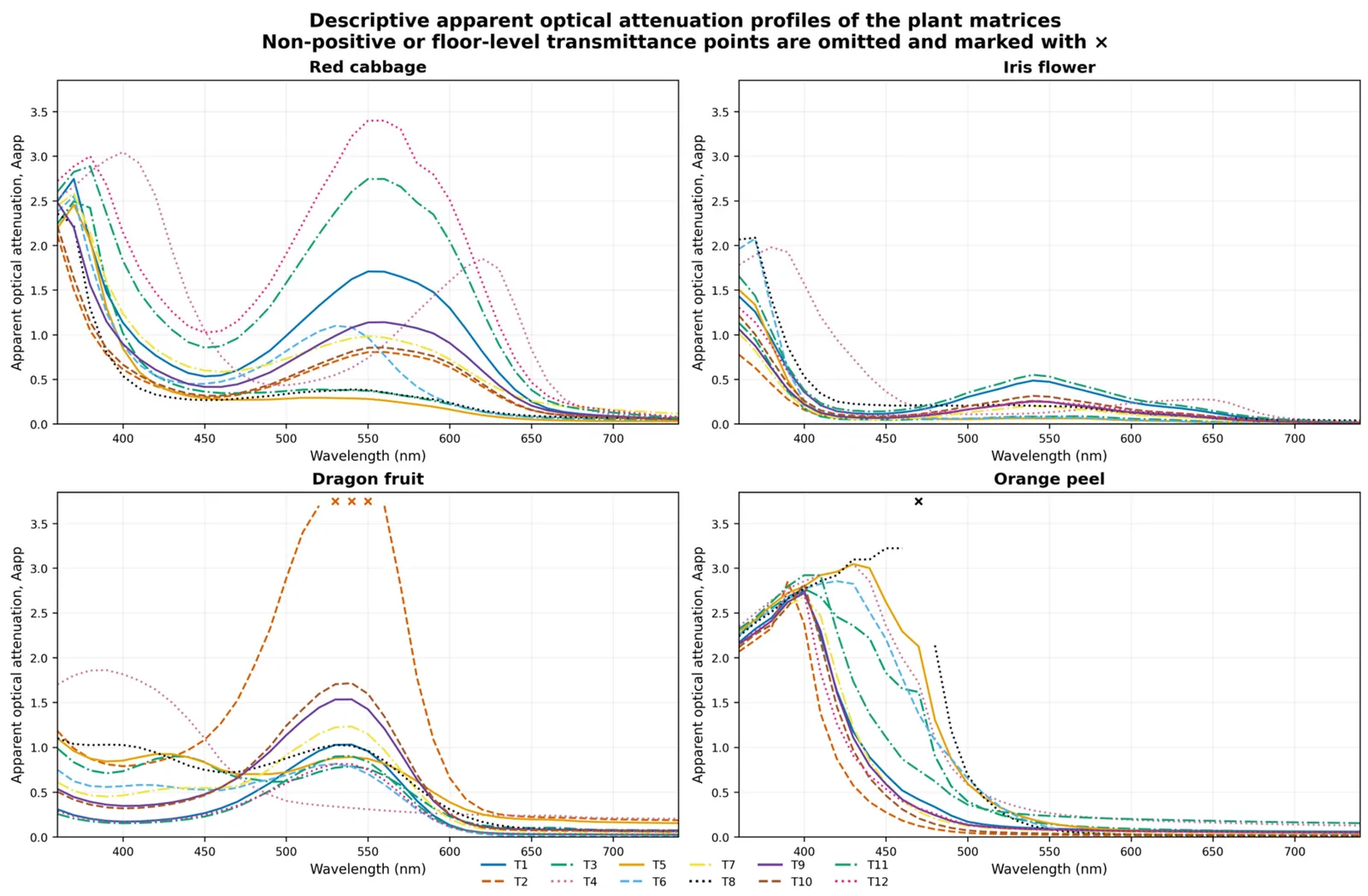
Descriptive apparent-optical-attenuation profiles of red cabbage, Iris flower, dragon fruit and orange peel under all twelve composite scenarios. Aapp is reported only for measured %T > 0.01; floor-level or non-positive points are omitted and marked with ×. The profiles may include absorption and scattering and are presented for qualitative shape comparison only, without score-based selection or quantitative ranking.

The dragon fruit records spanned magenta to yellow appearances. T2 had the lowest observed L* (34.06), while T10 had the highest observed C*ab (75.37). These values are reported separately and are not used to establish a rank. The T2 spectrum also contained three points at or below the 0.01%T operational floor, and treatment-specific filtration and dilution records were not recoverable. Consequently, neither the CIELAB extrema nor the attenuation profiles support quantitative within-matrix comparison. The green-region attenuation features are compatible only with a literature-informed betalain-like optical hypothesis for dragon fruit [9,18,23]. They do not resolve betanin, other betacyanins or any individual compound, and they cannot exclude contributions from other absorbing or scattering constituents. All dragon fruit interpretations are therefore limited to descriptive pigment-class compatibility. The complete profiles for the red-purple matrices and orange peel are presented in Figure 5.

### 2.3. Yellow-Orange and Brown Matrices

Orange-peel observations were dominated by positive b* values. T4 had the lowest observed L* (75.17), whereas T8 had the highest observed C*ab (126.18); these separate extrema do not identify a preferred extraction condition. T8 contained a censored transmittance point at 470 nm and is therefore excluded from quantitative attenuation indices. The general short-wavelength attenuation pattern is compatible with a literature-informed carotenoid-class hypothesis for citrus material [7,10,25,26]. The term carotenoid-compatible denotes class-level optical consistency only: the present data cannot distinguish molecular absorption from scattering, confirm that carotenoids caused the measured signal, identify any individual carotenoid or establish extraction yield.

**Figure 6:**
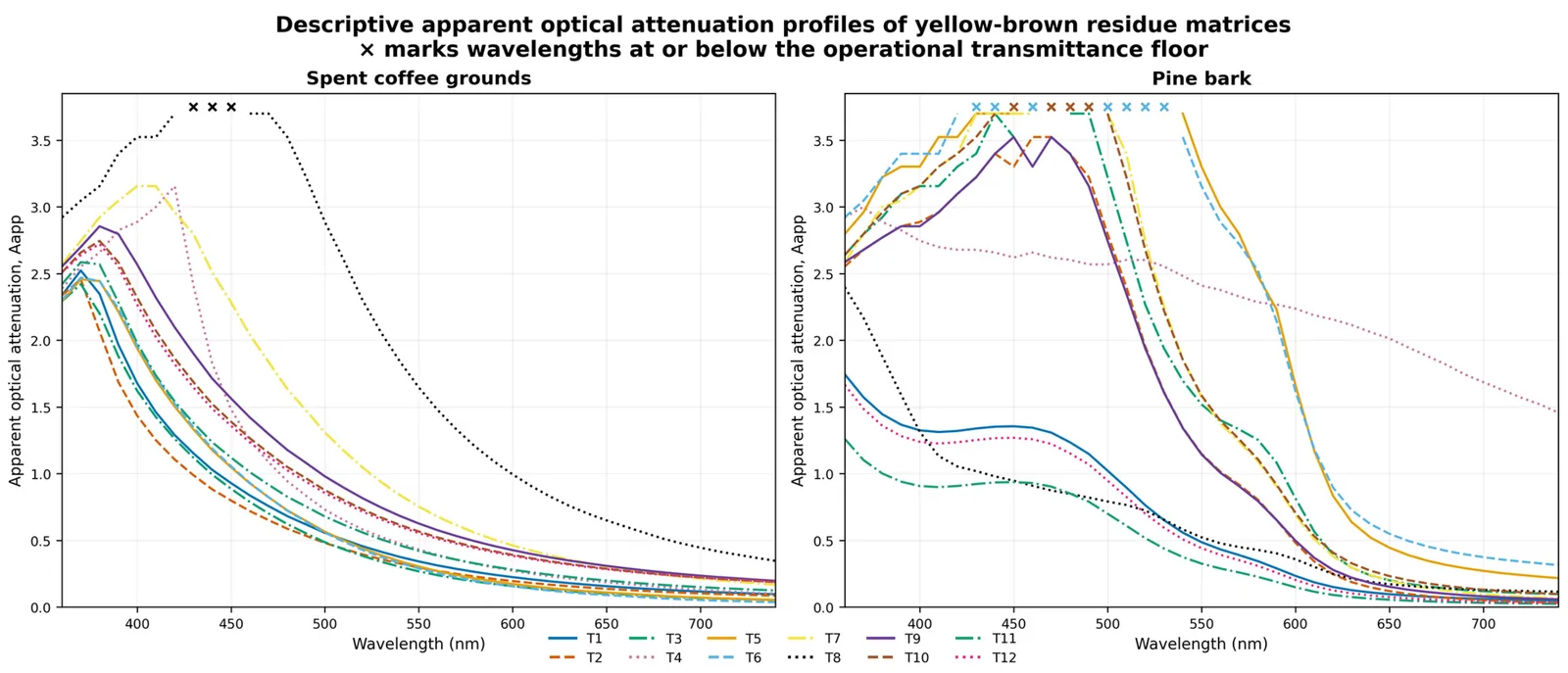
Descriptive apparent-optical-attenuation profiles of spent coffee grounds and pine bark under all twelve composite scenarios. Aapp is reported only where measured %T > 0.01; censored points are omitted and marked with ×. Broad attenuation may combine molecular absorption, light scattering and suspended-particle effects. The figure is qualitative and no treatment ranking is applied.

Spent coffee grounds and pine bark produced broad optical attenuation profiles rather than narrow pigment-specific features. For coffee, T8 had the lowest observed L* (25.32) and T7 the highest observed C*ab (77.04); T8 contained three points at or below 0.01%T and is not used for quantitative attenuation indices. For pine bark, T4 had the lowest observed L* (3.81) and T2 the highest observed C*ab (93.13), while several other pine-bark scenarios contained censored transmittance points. Extremely dark or broad responses may reflect suspended particles, scattering, oxidation or degradation in addition to chromophore absorption. The brown-yellow profiles are therefore described only as compatible with literature-reported melanoidin-like, tannin-related, proanthocyanidin-related or oxidised-phenolic contributions; no class is confirmed and no scenario is ranked. The complete spent-coffee-ground and pine-bark profiles are presented in Figure 6.

The *Pisolithus* dataset is not interpreted in the main plant-focused Results. Its complete optical profiles and CIELAB values are retained in Supplementary Figure S7 and Supplementary Data File S1 solely as a clearly identified non-plant comparison.

### 2.4. Observed Screening Patterns Across Matrices

**Figure 7:**
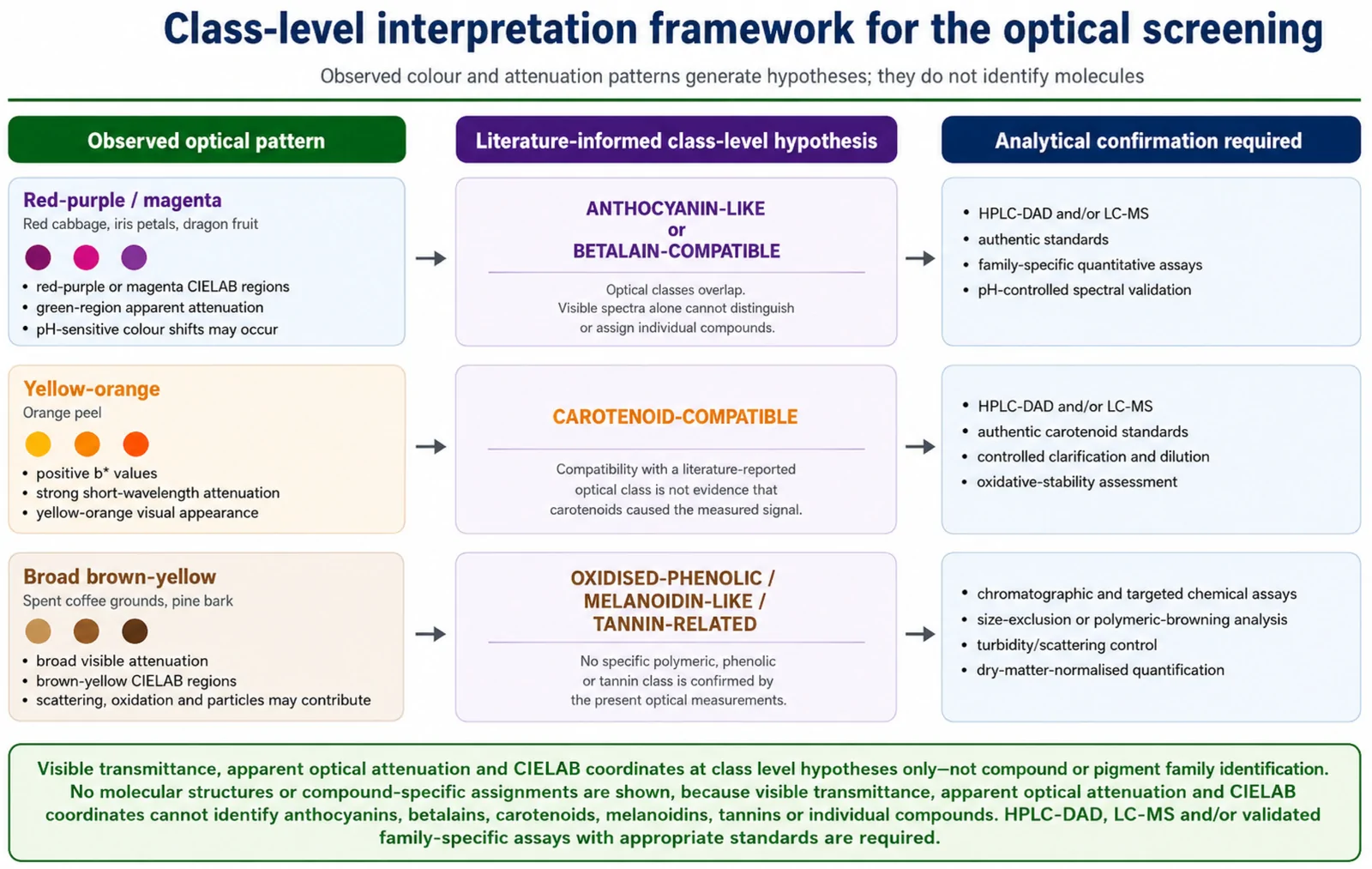
Class-level conceptual framework linking the observed CIELAB and apparent-optical-attenuation patterns to literature-informed hypotheses and the analytical confirmation required. The green boxes in the left column summarize the observed optical patterns, the purple boxes in the middle column show the literature-informed class-level hypotheses, and the blue boxes in the right column indicate the analytical confirmation required. The coloured row headings/text (e.g., red-purple/magenta, yellow-orange, and broad brown-yellow) visually correspond to the dominant observed colour families discussed in the manuscript. The arrows indicate the conceptual progression from the empirical optical observations to the provisional class-level interpretation and, finally, to the analytical methods needed for confirmation; they do not imply direct proof or compound identification. No molecular structures or compound-specific assignments are shown, because visible transmittance, apparent optical attenuation and CIELAB coordinates cannot identify anthocyanins, betalains, carotenoids, melanoidins, tannins or individual compounds. HPLC-DAD, LC-MS and/or validated family-specific assays with appropriate standards are required.

Across the single preparations tested, independent CIELAB extrema and qualitative attenuation shapes varied among matrices and composite scenarios, but no scenario is designated as best or highest-ranked. Red cabbage, Iris and dragon fruit displayed red-purple or magenta optical regions; orange peel displayed yellow-orange coordinates; and coffee and pine bark showed broad brown-yellow attenuation. These patterns generate hypotheses for future replicated work but do not demonstrate superior pigment recovery, isolated effects of freezing, ultrasound, heating, water, HCl/KOH addition or solvent choice, chemically validated pigment concentration, or the presence of a specific pigment family. Figure 7 has therefore been redesigned as a general class-level conceptual scheme linking observed optical patterns to literature-informed hypotheses and the chromatographic or targeted analyses required for confirmation; it contains no molecular structures or compound-specific assignments.

These observed patterns motivate matrix-specific hypotheses for independently replicated follow-up experiments rather than prescribing source-specific extraction conditions. These descriptive observations motivate the hypothesis that a single protocol may yield different optical appearances across pH-sensitive anthocyanin-like, carotenoid-compatible, betalain-compatible and broad brown-phenolic systems. This hypothesis has not been tested inferentially and requires independently replicated extractions before any process recommendation is made.

Because transformation and degradation were not independently controlled, differences among scenarios are interpreted only as endpoint optical differences. They cannot be partitioned into improved release, solubilisation, acid–base conversion, hydrolysis, saponification, oxidation, condensation, polymerisation or degradation, and none of these processes is quantified by the present dataset.

## 3. Discussion

The combined photographs, CIELAB coordinates and transmittance profiles provide a first-pass description of measured sample appearance. Aapp is used only as an operational optical-attenuation transform for positive transmittance values above 0.01%T. Because clarification, turbidity, sedimentation and conventional UV–visible validation were not standardized, the measured attenuation may combine molecular absorption and light scattering and is not used for quantitative treatment comparison, pigment concentration, yield or purity. The revised analysis therefore reports CIELAB dimensions and optical-profile shapes separately and does not use a composite score or treatment rank. Visible optical profiles and photographs can suggest pigment-class hypotheses, but they cannot replace clarified, diluted and conventionally validated UV–visible measurements, HPLC-DAD, LC-MS or targeted assays. In this context, terms such as anthocyanin-like, betalain-compatible, carotenoid-compatible, melanoidin-like and pulvinic-acid-type are used solely as optical-class descriptors and do not confirm the presence of any specific molecule or pigment family in the extracts. Accordingly, the principal contribution is a proof-of-concept screening workflow that documents a broad matrix–scenario space and identifies observations for follow-up work; it does not establish process optima, quantitative extraction yields or statistically validated superiority among treatments. Methodologically, the twelve conditions should be understood as composite practical scenarios rather than a causal design: the present workflow can document complete scenario records, but it cannot rank extraction performance or estimate isolated effects of solvent, time, temperature, freezing, ultrasound or acid/base addition. The revised conceptual scheme therefore stops at class-level hypotheses and explicitly separates optical observation from chemical identification.

Interpretive framework: Direct observations in the following discussion are restricted to the recorded photographs, CIELAB coordinates and qualified spectral shapes. Statements concerning cell disruption, cavitation, acid–base equilibria, saponification, oxidation, condensation, pigment chemistry or degradation are literature-informed hypotheses only. None of those mechanisms was directly measured, and they are not used to infer extraction efficiency, pigment concentration or causal treatment effects.

The red-purple plant matrices were consistent with anthocyanin-like or betalain-compatible behaviour, but the two chemistries must be separated mechanistically. Red cabbage is the clearest anthocyanin-like case. Red cabbage extracts are dominated in the literature by cyanidin-based glycosides, often acylated with hydroxycinnamic acids, and selected cultivars show λmax values around 520 nm under acidic conditions and approximately 610 nm at neutral pH when diacylated blue-colour fractions are enriched [16,17]. These literature-reported features are optically compatible with the present T11/T12/T1 profiles and the KOH-added T4 bathochromic displacement, but they do not identify the compounds responsible for those profiles. The literature describes tissue freezing as disrupting cellular compartments and acoustic cavitation as increasing mass transfer; in the present study, these mechanisms remain possible explanations only and do not demonstrate that freezing or ultrasound increased extraction or produced a superior treatment response. However, the KOH-associated colour should not be interpreted as superior extraction quality for food applications; it may reflect a pH-dependent change in an anthocyanin-like chromophore state, with potentially reduced stability and different regulatory/application implications. The absence of measured pH prevents assignment of this displacement to a defined anthocyanin equilibrium state. The references to cyanidin-derived glycosides and anthocyanin equilibria describe published red-cabbage chemistry only and must not be read as assignment of those compounds or equilibria to the present extracts. The proposed freezing-, ultrasound- and pH-related explanations are therefore alternative literature-based interpretations, not mechanisms demonstrated by the present endpoint measurements; chromophore transformation or degradation cannot be excluded.

Dragon fruit illustrates why pigment-family specificity matters. Its magenta CIELAB position and apparent-optical-attenuation feature near the green region supports only a literature-informed betalain-compatible optical hypothesis. The present data cannot distinguish betacyanins from other absorbing or scattering contributors and do not identify betanin or any other individual compound. Reported visible maxima around 535–540 nm and the known pH-dependent degradation chemistry of betalains are cited solely as background [9,18,23,27], not as molecular confirmation of the measured extracts. Within the available dragon fruit CIELAB records, T2 had the lowest L* while T10 had the highest C*ab; neither extremum can be interpreted as a valid rank or treatment effect because filtration and dilution were not standardised or traceable, turbidity was not measured and T2 contained censored transmittance points. For a food-colourant application, dragon fruit would merit replicated mild aqueous extraction, immediate pH control and stability testing rather than intensification by harsher processing. No parent-pigment/degradation-product profile or time-course control was obtained, so the observed differences cannot distinguish extraction from betalain transformation, oxidation or degradation.

The yellow-orange and brown matrices represent a different extraction problem. Orange peel is treated only as a carotenoid-compatible optical hypothesis rather than as a chemically identified carotenoid extract. The high b* and strong short-wavelength attenuation are compatible with short-wavelength behaviour reported for carotenoid-containing citrus material [7,10,25,26], but this correspondence does not identify carotenoids or demonstrate that they caused the measured signal. The observed T4 colour is compatible with effects that KOH-added media can produce, including saponification of carotenoid esters and membrane disruption; nevertheless, this optical response does not confirm carotenoid identity or a specific reaction pathway, and it is not automatically desirable. Strong alkali and acetone-containing treatments are poor scenarios for direct clean-label development. A more publishable translational route would be to use the present optical screening to justify replicated tests with ethanol, food-grade oils, limonene, glycerol-based natural deep eutectic solvents or emulsion-assisted extraction, followed by HPLC carotenoid profiling and oxidative stability testing. This mechanistic interpretation is provisional because the initial and final pH values were not measured. The T8 optical profile contains a censored transmittance point and is not used for quantitative spectral interpretation. In particular, the current data do not distinguish increased carotenoid release from alkali- or heat-induced chemical transformation, oxidation or degradation.

Coffee grounds and pine bark should be discussed as broad-absorbing mixtures rather than single-pigment sources. In coffee, caffeine may be analytically abundant and chemically recognisable, but it is not the main cause of the brown colour because caffeine absorbs primarily in the ultraviolet region near 272 nm rather than across the visible range [28]. The broad visible colour is more cautiously regarded as compatible with possible contributions from melanoidin-like material, chlorogenic-acid derivatives, quinone-like species and other oxidised phenolics reported in coffee, rather than as confirmation of any one class. Literature commonly uses A420 as a browning/melanoidin indicator, and melanoidin spectra decrease broadly with increasing wavelength [11,12,14,19]. The similarly broad pine-bark response is compatible with possible contributions from tannin-, proanthocyanidin- and phenolic-oligomer-related components; oxidation and condensation of such literature-reported components can produce visible yellow-brown species even when the parent flavan-3-ols have stronger UV than visible absorption [13,20]. In the present study, A420 and other Aapp descriptors are treated as qualitative optical-attenuation indicators only; without turbidity control and conventional UV–visible validation they cannot be assigned specifically to melanoidins, tannins or pigment concentration. The class labels used for these broad mixtures are therefore descriptive hypotheses only and do not identify melanoidins, tannins, proanthocyanidins, quinones or other specific constituents. Likewise, the dataset cannot separate pre-existing brown chromophores from oxidation, condensation or polymerisation occurring during processing or storage; these pathways remain untested hypotheses.

The principal analytical constraints have been detailed in the Materials and Methods and Results. In brief, the single-preparation design supports descriptive observations only, not treatment ranks, statistically significant differences or isolated process effects. No simulated pH values were introduced; dragon fruit comparisons remain limited by untraceable filtration and dilution; turbidity and scattering were not independently controlled; and 35 measurements at or below 0.01%T are treated as censored rather than forced to Aapp = 4.00. The previous composite score and “highest-ranked” terminology were removed. The complete validation pathway, independent replication, dry-matter normalisation, a controlled factorial or sequential design, standardised clarification, conventional UV–visible cross-validation, complete process metadata and transformation/degradation controls, is consolidated in the Conclusions.

Taxonomic and provenance incompleteness constitutes an additional source of biological uncertainty because pigment profiles may vary with species, cultivar, organ, maturity, growing environment and post-harvest history. The present optical responses therefore cannot be extrapolated beyond the particular batches tested. A future seasonal sampling campaign should prospectively record the supplier or georeferenced collection locality, collection date, cultivar where applicable, developmental stage, exact plant or fungal part, pre-treatment and storage history, and should deposit herbarium or fungarium vouchers. Ambiguous plant and fungal materials should be confirmed by an appropriate taxonomic expert and, where necessary, by DNA barcoding before species-level comparisons are made.

## 4. Materials and Methods

### 4.1. Biological Materials and Reagents

Six plant/agro-food materials formed the main dataset: orange peel (*Citrus sinensis*), spent coffee grounds (*Coffea arabica*), red cabbage (*Brassica oleracea* var. *capitata* f. *rubra*), dragon fruit (*Hylocereus*/*Selenicereus* sp.), *Iris germanica* petals and pine bark (*Pinus* sp.). A *Pisolithus* sp. sample was analysed in the original campaign but is excluded from the main-text comparisons and retained only as a supplementary non-plant comparator. Materials were obtained locally or by direct collection and stored at 2 °C before extraction. Reagents included distilled water, ethanol, acetone 30%, potassium hydroxide (KOH) 30%, hydrochloric acid (HCl)^−^ 1 N and glacial acetic acid. The surviving laboratory records use “30%” as a nominal preparation label. For T4, 100 µL of the stock labelled “30% KOH” was added to a 20.0 mL extraction bath, corresponding to a 0.50% (*v*/*v*) stock-solution addition. The original record does not specify whether the 30% KOH stock was prepared on a *w*/*w* or *w*/*v* basis and does not retain its density; therefore, an exact final KOH mass or molar concentration cannot be reconstructed without inventing information. For T8, the working medium was recorded only as “30% acetone”; the percentage basis and preparation volumes were not preserved, so it is retained as a nominal laboratory designation rather than reported as an exact final concentration. By contrast, the recoverable compositions are unambiguous for T5 and T6: T5 contained 100 µL of 1 N HCl in a 20.0 mL bath (nominal acid-equivalent concentration 0.005 N), and T6 contained 16 mL ethanol plus 4 mL glacial acetic acid (80:20, *v*/*v*; 20% *v*/*v* glacial acetic acid). All commercially purchased chemical reagents used in this study were supplied by Sigma-Aldrich (Merck KGaA, Darmstadt, Germany).

The taxonomic names reported above reproduce the identifications available in the original source labels and laboratory records and should not be interpreted as voucher-confirmed determinations. The precise species remains unresolved for the dragon fruit material (*Hylocereus*/*Selenicereus* complex), pine bark (*Pinus* sp.) and the supplementary *Pisolithus* comparator. Orange peel, spent coffee grounds, red cabbage and *Iris* petals were recorded at the source-matrix level, but cultivar, developmental stage, exact collection locality and date, and voucher number were not systematically documented. The material part or processed form was explicit only where encoded in the matrix label (orange peel, spent coffee grounds, *Iris* petals and pine bark); it was not separately recorded for red cabbage, dragon fruit or the *Pisolithus* material. No herbarium or fungarium vouchers were deposited. Because the original batches were consumed and several materials are seasonal or perishable, these missing metadata cannot be reconstructed retrospectively. Consequently, the results are restricted to the specific source batches and matrix categories tested and must not be generalized to a taxonomically validated species, cultivar or developmental stage.

Because the screened materials differed substantially in water content and tissue structure, the comparison is interpreted primarily within each matrix. Direct yield comparison among matrices should be avoided unless future experiments normalise extractions on a dry-matter basis. All nominal 0.2 g portions were weighed on an as-received basis. Moisture content and dry-matter fraction were not measured; consequently, the solid-to-liquid ratio is an as-received-mass ratio and not a dry-matter-normalised ratio. Cross-matrix differences in apparent optical attenuation or CIELAB coordinates therefore describe only the optical appearance of the measured extracts and cannot be interpreted as comparative extraction efficiency, pigment yield or pigment concentration.

**Figure 8:**
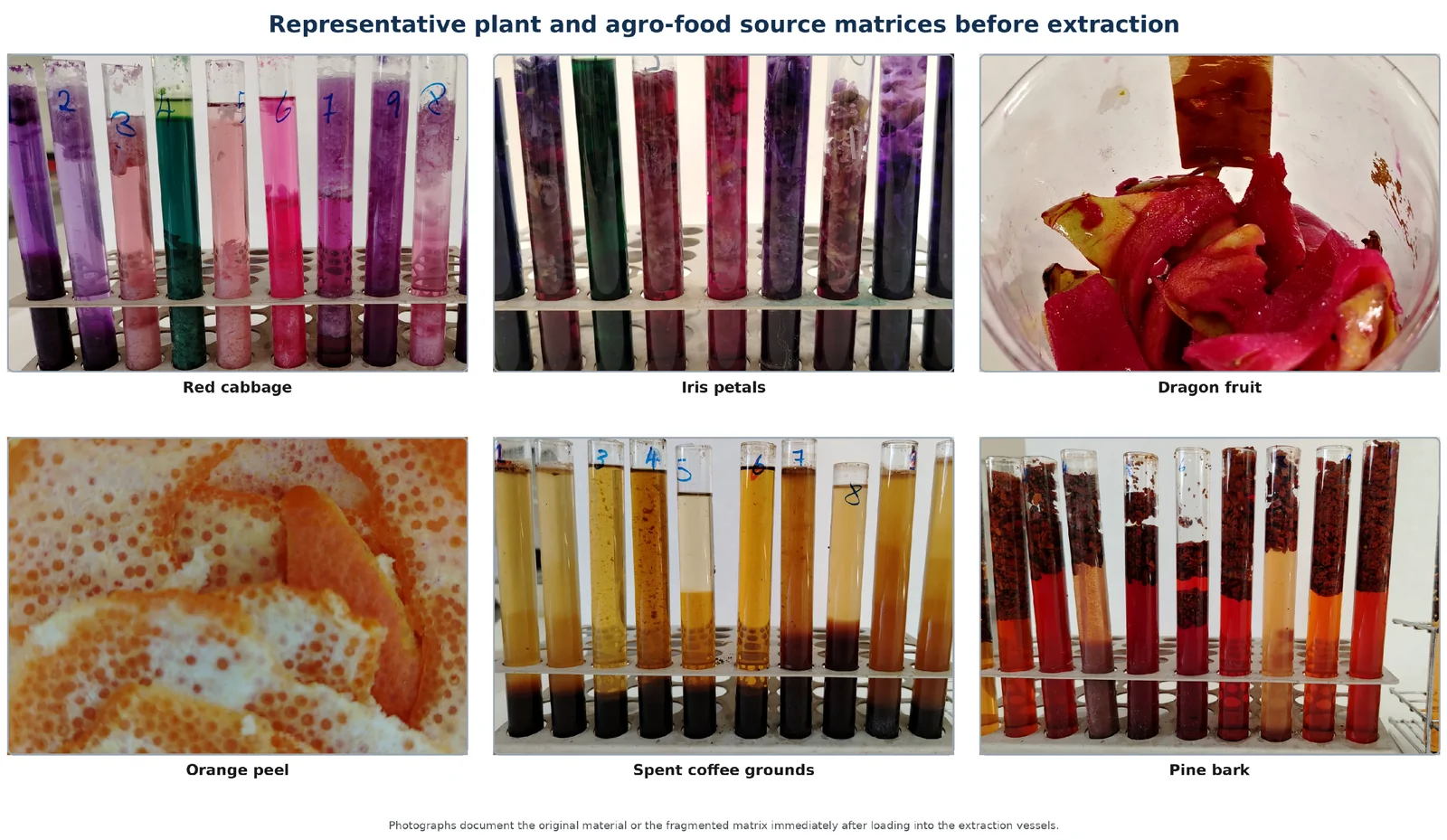
Representative plant and agro-food source matrices used in the main screening: red cabbage, *Iris* petals, dragon fruit, orange peel, spent coffee grounds and pine bark. The images document the original material or the fragmented matrix immediately after loading into the extraction vessels and are not intended for quantitative colour evaluation.

Representative photographs of the six plant/agro-food source matrices before extraction, or immediately after manual fragmentation and loading into the extraction vessels, are provided in Figure 8 to document the physical materials used in the screening.

### 4.2. Sample Preparation and Composite Screening Design

Samples were superficially cleaned, manually fragmented and weighed before extraction. Soft matrices such as dragon fruit, red cabbage and petals were pre-crushed to facilitate pigment release, whereas more resistant materials were ground with a mortar to increase contact area. Approximately 0.2 g of material was used per treatment with a final extraction volume of 20 mL, except where a defined solvent mixture was required. The twelve extraction treatments are summarised in Table 3. The nominal 0.2 g/20 mL combination corresponds to a 1:100 solid-to-liquid ratio (*w*/*v*). It was selected as a standardized screening compromise that provided sufficient liquid volume for complete wetting and mixing of the heterogeneous matrices, transfer to a 10 mm cuvette and filtration or dilution when required, while limiting consumption of biological material and extraction medium. The relatively high liquid-to-solid ratio also reduced the likelihood that highly concentrated or turbid extracts would exceed the useful optical range of the transmittance measurement. The same nominal ratio was maintained across matrices to support within-study comparability; it was not intended to represent a source-specific optimum and should be re-evaluated on a dry-matter basis during subsequent replicated optimization. The 0.2 g input mass was not corrected for matrix moisture. This limitation is especially relevant when fresh fruit, petals and cabbage are considered alongside drier coffee-ground and bark materials; a valid yield comparison will require gravimetric moisture determination and reporting per gram of dry matter in a future replicated campaign.

Treatments T1 and T2 were aqueous extractions for 45 min, differing by prior freezing. Treatments T3 to T8 combined heating at 70 °C or boiling water with different solvent or acid/base-reagent conditions. Treatments T9 to T12 used an ultrasonic bath for 10 min, varying temperature and prior freezing. Initial and final pH were not measured; therefore, T4 and T5 are described only as KOH-added and HCl-added scenarios, respectively, and no numerical pH value or controlled-pH status is assigned. The longer 45 min contact time used for T1 and T2 was chosen because these treatments were passive room-temperature aqueous extractions and therefore received no mass-transfer assistance from heating or ultrasound. Ultrasound-assisted extractions were performed using a 1.5 L Ultrasons-HD series ultrasonic bath (J.P. Selecta S.A.U., Abrera, Barcelona, Spain), supplied through Fisher Scientific S.L. (Alcobendas, Madrid, Spain). The additional contact time was intended to allow tissue hydration and diffusion-driven release of water-soluble colour-bearing compounds while retaining a low-energy extraction route. By contrast, T3–T8 used heating at 70 °C or boiling water, and T9–T12 used ultrasound, so a 10 min treatment was adopted to limit thermal or sonochemical exposure while taking advantage of the faster mass transfer provided by those processes. These durations were fixed screening conditions rather than the result of a kinetic optimization and should be investigated systematically in future time-course experiments.

Reproducibility-metadata audit: The surviving laboratory records preserve the nominal input mass (approximately 0.2 g) and final extraction volume (20 mL), but not the exact mass recorded for each individual preparation. Fragmentation was manual or performed with a mortar; no sieve fraction, particle-size range or particle-size distribution was measured. Prior freezing is identified in the treatment code, but freezing duration and temperature were not preserved. Ultrasound-assisted scenarios were performed in an ultrasonic bath, but the bath model, nominal frequency, electrical or delivered acoustic power, and sample position within the bath were not recorded. The extraction-vessel material, geometry, fill volume/headspace and agitation conditions during passive or heated treatments were also not documented. Source materials were stored at 2 °C before extraction; however, post-extraction storage duration and temperature, vessel closure and headspace, protection from ambient light, and elapsed time between extraction, clarification and optical measurement were not systematically recorded. Clarification limitations are detailed in Section 4.3. These details cannot be reconstructed retrospectively and their absence prevents exact independent reproduction of the original campaign. A future protocol must record, for every preparation, exact mass, particle-size specification, freezing conditions, ultrasound frequency and delivered power, vessel geometry, agitation, clarification, oxygen/light exposure, storage and time-to-measurement.

The twelve treatment codes represent composite extraction scenarios rather than a controlled factorial or one-factor-at-a-time design. Solvent, contact time, temperature, freezing, ultrasound and acid/base addition were combined to survey a broad practical screening space. Consequently, the present data cannot assign a main effect or interaction to any individual variable, and comparisons are restricted to complete scenario-wise observations. A follow-up validation study should use independent extraction replicates within each matrix and either a controlled factorial design or a sequential one-factor-at-a-time design with explicit baseline conditions and appropriately matched times and temperatures.

Retrospective deduction or simulation of final pH was not undertaken. Fixed HCl or KOH additions cannot predict final pH in heterogeneous biological matrices because buffering capacity, native acidity, water content, solvent composition and temperature differ among samples. Reporting calculated values would therefore create unsupported data rather than recover a missing measurement. The original extracts are no longer available for direct measurement. Future replicated experiments will record pH of the extraction medium before reagent addition, immediately after addition, after extraction, and after any filtration or dilution, using a calibrated electrode appropriate to the solvent composition and measurement temperature. The composition labels in Table 3 have therefore been revised to distinguish measured volumes and nominal stock additions from concentrations that cannot be recovered retrospectively. T6 is no longer described as an “acidified hydroalcoholic medium”; it is explicitly identified as a concentrated 80:20 (*v*/*v*) ethanol–glacial acetic acid mixture with no intentionally added water. This composition is chemically and operationally distinct from mild acidification of an aqueous or hydroalcoholic solvent.

### 4.3. Spectrophotometric and Colorimetric Measurement

After extraction, liquids were transferred to 10 mm glass cuvettes. Turbid or mucilaginous dragon fruit extracts were filtered and diluted with the corresponding solvent to obtain homogeneous samples suitable for transmittance measurement. Visible transmittance spectra and CIELAB coordinates were measured using a CM-36dG benchtop spectrophotometer (Konica Minolta, Inc., Sensing Business Unit, Sakai, Osaka, Japan) over the 360–740 nm wavelength range. The corresponding extraction solvent was used as blank for baseline correction. The instrumental configuration used the specular component included mode and a large-area view setting; colorimetric measurements were processed under D65/10° conditions. Re-examination of the surviving records did not identify which individual dragon fruit treatments were filtered or diluted, the filter material or nominal pore size, the exact dilution factors, or whether the reported spectra were back-corrected to the original concentration. These details cannot be reconstructed from the available files. Accordingly, the dragon fruit spectra and CIELAB values are retained for transparent qualitative description of a betalain-compatible optical response, but they are excluded from formal treatment ranking and quantitative within-matrix comparison. No standardized clarification or centrifugation procedure was applied across all matrices, and turbidity, suspended-particle concentration and time-dependent sedimentation were not measured. The CM-36dG transmittance signal therefore represents total optical attenuation and cannot separate molecular absorption from light scattering. No validation against a conventional cuvette UV–visible spectrophotometer was performed. Because the original extracts and source batches are no longer available, these controls could not be added retrospectively during revision; they are required in a replicated follow-up study.

Measured transmittance spectra reported as %T were converted to decimal transmittance according to τ(λ) = %T(λ)/100. Apparent optical attenuation was calculated as Aapp (λ) = −log10[τ(λ)] only when the measured value exceeded 0.01%T. In the revised processing, values at or below 0.01%T are treated as censored and left untransformed; they appear as gaps and × markers in the revised figures, and no artificial Aapp ceiling of 4.00 is used. Thirty-five wavelength points across nine matrix–scenario combinations met this censoring criterion. Samples containing censored points are excluded from aggregate apparent-attenuation indices and from quantitative spectral comparison. The original measured %T values remain unchanged. Aapp is an operational optical-attenuation transformation and must not be interpreted as calibrated pigment concentration or as pure molecular absorbance. Complete spectroscopic documentation is provided for every matrix and treatment: Supplementary Figures S1–S6 present paired measured transmittance and derived apparent-optical-attenuation profiles for the 72 plant/agro-food matrix–scenario combinations in the main analysis (6 matrices × 12 scenarios; 39 wavelengths per spectrum). Supplementary Figure S7 and the corresponding rows in Data File S1 retain the 12 *Pisolithus* combinations solely as a non-plant supplementary comparison.

Photographs of selected liquid extracts were retained from the original screening record as qualitative documentation of visible hue differences. These photographs were not processed for quantitative image-based colour analysis.

Chromophore-transformation controls were not included in the original screening. No matched untreated, dark, oxygen-excluded or time-zero controls, no time-resolved spectra, and no chromatographic mass balance were used to distinguish pigment release from chemical conversion or degradation. Heating, concentrated acid or alkali, ultrasound, freezing, oxygen exposure, ambient light and delay before measurement may each alter chromophore state or generate degradation/oxidation products. Consequently, every reported spectrum must be regarded as the net endpoint of solubilisation/release, scattering and any transformation or degradation that occurred under the composite scenario, rather than as a direct measure of extraction efficiency. Future validation should include matched process controls, controlled light and oxygen exposure, pH and temperature histories, time-course measurements, and HPLC-DAD and/or LC-MS analysis of parent pigments and transformation products.

CIELAB coordinates were calculated according to the CIE L*a*b* framework under D65/10° conditions [29,30]. CIELAB chroma (C*ab) was calculated using the standard expression C*ab = √[(a*)^2^ + (b*)^2^]. The hue angle was reported as °Hue (standard CIELAB symbol h°ab) and calculated in degrees as °Hue = atan2(b*, a*) × 180/π; when the initial result was negative, 360° was added so that values were expressed within 0° ≤ °Hue < 360°. In the remainder of the manuscript, “chroma” is used as the descriptive term, while C*ab is retained as the standardized CIELAB symbol in equations, tables and figures. L*, a*, b*, C*ab and °Hue are reported as separate descriptive dimensions. No composite colour score is calculated. Descriptive Aapp point values and aggregate attenuation indices are retained in Supplementary Data File S1 only for uncensored records; they are not used to rank treatments or to infer extraction yield, pigment concentration, purity or application performance.

### 4.4. Descriptive Colorimetric Reporting and Statistical Considerations

The previous intensity-oriented score that combined normalized low L* and high C*ab with equal weights has been removed because it was not validated against chemical pigment measurements or application performance. No composite index, selection-status label or treatment rank is used in the revised analysis. L*, a*, b*, C*ab and °Hue are interpreted independently as coordinates of measured sample appearance. Table 2 reports separate observed extrema for L* and C*ab solely to describe the data range; these extrema do not identify an extraction optimum or a preferred treatment.

Each matrix–treatment combination was prepared once. Consequently, no experimental variance could be estimated, and no inferential statistical analysis was applied. Results are interpreted as descriptive trends under the tested conditions rather than statistically significant treatment differences.

The single-preparation strategy was deliberately adopted to maximise breadth at the proof-of-concept stage, covering 72 plant/agro-food matrix–scenario combinations in the main analysis, while 12 *Pisolithus* combinations were retained only as a supplementary comparator before defining observations for replicate-based validation. Accordingly, the dataset is valid as an internally standardised descriptive record of the specific preparations tested, rather than as an estimate of population-level treatment effects. Internal comparability was supported by applying the same nominal solid-to-liquid ratio, blanking procedure, cuvette path length, spectral range, instrument settings, illuminant/observer conditions and data-processing workflow to all treatments. The 39 wavelength values within each spectrum describe the optical profile and were not treated as independent experimental replicates. Consequently, this design cannot quantify between-preparation variability, support inferential statistics, demonstrate reproducibility or establish statistically validated treatment superiority. Its experimental validity is therefore restricted to objective instrumental screening and descriptive identification of observations for follow-up; the documented observations require confirmation through independently replicated extractions and an appropriate pre-specified statistical design. Before any treatment effect is inferred, follow-up validation must include a minimum of three independently prepared extraction replicates per matrix–treatment combination, with the final sample size increased if required by variance estimates and an a priori power analysis. Independent extraction replicates could not be generated during the present revision because the original source batches had been exhausted and several plant and fungal matrices are seasonal and perishable. Collecting replacement material outside the original availability window would introduce a new biological batch and season rather than reproduce the original extraction campaign. Replicated validation is therefore planned for the next appropriate seasonal availability period, with independent batches, collection dates and provenance metadata recorded prospectively.

### 4.5. Software and Data Processing

Spectral transformations, descriptive calculations and data-derived plots were performed using Python 3.13 (Python Software Foundation, Beaverton, OR, USA) and Matplotlib 3.10.8 (Matplotlib Development Team).

## 5. Conclusions

Visible spectroscopy, apparent-optical-attenuation transformation and CIELAB colorimetry provided a practical first-pass framework for describing the optical appearance of extracts from chemically diverse matrices. The results support a cautious, putative pigment-class interpretation based on optical compatibility rather than direct molecular identification. Within the single preparations tested, separate extrema of L* and C*ab varied among scenarios and were not consistently produced by the same treatment. No best treatment or composite rank is assigned. Dragon fruit observations remain non-comparable because filtration/dilution history was not standardised, and spectra containing floor-level transmittance are treated as censored rather than quantitatively interpreted. These observations do not establish that any treatment favoured, preserved or intensified pigment recovery. Coffee grounds and pine bark showed broad brown-yellow profiles compatible with possible contributions from melanoidin-like, oxidised-phenolic, tannin-related and proanthocyanidin-related components. Their observed optical profiles are more consistent with broad brown/amber appearances than with narrow-hue colorants; this is a descriptive colour-space interpretation, not evidence of superior application performance. The non-plant *Pisolithus* comparison is reported only in the Supplementary Materials and is not included in the plant-focused conclusions. Overall, this study documents batch-specific matrix–scenario observations and mechanistic hypotheses for replicated extraction, chromatographic pigment identification, pH-controlled stability testing and application-specific validation. No specific compound or pigment family is confirmed by the present visible spectroscopy and CIELAB data; all class-level assignments remain hypotheses requiring chromatographic or molecular verification. In this proof-of-concept context, the separate CIELAB extrema and qualified optical profiles describe the conditions tested rather than validated extraction performance. The principal contribution is therefore a transparent descriptive screening record that provides a basis, not a substitute, for replicated optimisation, quantitative yield determination, chromatographic confirmation and application-specific validation. Because extraction yield, recovery percentage, mass balance, purity, pigment concentration and application performance were not determined, neither the title nor the conclusions describe colorant recovery as a measured result. The redesigned class-level scheme contains no chemical structures and does not assign any molecule or pigment family. The spectral differences are net endpoint observations and may include chromophore transformation or degradation; they do not demonstrate the mechanistic explanations discussed from the literature.

The methodological limitations also determine the interpretation and transferability of the present results. The single preparation of each matrix–treatment combination prevents estimation of experimental variability and inferential comparison; the absence of dry-matter-normalised yields precludes quantitative comparison of extraction efficiency among matrices; and the lack of molecular analysis and application-specific stability testing prevents compound-level identification and performance claims. A staged future research pathway should therefore: (i) repeat each extraction in independent replicates and analyse the data using an appropriate pre-specified statistical design; (ii) normalise recovery to dry matter and systematically evaluate pH, temperature, time, solvent composition and solid-to-liquid ratio; (iii) identify and quantify individual pigments using HPLC-DAD and/or LC-MS, supported by family-specific assays where appropriate; and (iv) assess the stability of the selected extracts under conditions representative of intended uses, including controlled exposure to light, oxygen, pH, temperature, storage and the relevant food, packaging, coating or textile matrix. Until these steps are completed, the present results should be interpreted as screening priorities for follow-up experimentation rather than as validated process optima, quantitative yield comparisons, confirmed pigment identities or demonstrated application stability. For the replication step, at least three independent extraction preparations per retained matrix–treatment combination are required as a minimum, while the definitive sample size should be justified from pilot variance and power considerations. The observations reported here must therefore be understood as batch-specific matrix observations rather than as validated species-, cultivar- or developmental-stage effects. In addition, the screening scenarios must be replaced by a controlled matrix-specific factorial or sequential design before any individual extraction-variable effect is claimed. A future dry-matter-normalised design must determine moisture content for each biological lot and report recovery per gram of dry matter. It must also predefine a uniform clarification protocol or record, for every sample, filter material and pore size, dilution factor and the mathematical back-correction applied before any quantitative comparison or process selection is made. The validation stage must additionally apply a uniform clarification/centrifugation or filtration protocol, record turbidity and sedimentation, dilute every sample into the verified linear range, treat values below the quantifiable transmittance limit as censored, and cross-check representative spectra using a conventional UV–visible spectrophotometer. The validation protocol must additionally standardise and report all currently missing process metadata and include dark, oxygen-controlled, time-zero and time-course controls together with chemical profiling of parent pigments and degradation products.

## Figures and Tables

**Table 1:** Measured CIELAB a* and b* coordinates for all twelve treatments within each of the six plant/agro-food matrices. Each cell is reported as a*/b*. Positive a* denotes the red direction and negative a* the green direction; positive b* denotes the yellow direction and negative b* the blue direction. The Pisolithus comparator is excluded from this main-text table and remains available in Supplementary Data File S1.

Treatment	Red Cabbage	*Iris* Flower	Dragon Fruit	Orange Peel	Spent Coffee Grounds	Pine Bark
T1	40.58/−37.89	23.69/−23.35	62.86/−20.91	−9.88/57.27	9.19/51.36	29.38/65.90
T2	22.71/−26.95	14.91/−14.11	68.52/1.22	−10.20/32.71	7.27/46.98	53.92/75.93
T3	15.04/9.44	3.00/−2.44	34.68/25.45	−0.25/99.67	8.62/55.81	53.38/58.50
T4	−55.07/9.74	−22.97/24.22	−4.54/55.38	4.25/95.49	13.36/69.52	3.57/2.98
T5	11.89/9.39	1.61/2.19	31.71/15.39	8.13/112.02	11.20/62.11	47.84/29.97
T6	52.60/−4.15	2.34/1.48	48.11/13.73	10.79/110.73	11.82/64.13	44.47/27.10
T7	23.59/−10.49	10.77/−10.61	59.40/−1.78	−10.78/52.12	26.19/72.45	54.76/61.53
T8	16.44/−2.04	6.80/5.02	45.75/15.43	8.56/125.89	33.68/43.23	21.05/37.65
T9	28.55/−31.48	13.18/−12.83	69.27/−15.37	−10.71/50.92	17.34/59.20	51.72/74.71
T10	23.57/−27.87	15.71/−15.44	73.13/−18.23	−12.08/46.35	14.95/56.41	53.24/59.95
T11	44.55/−31.57	9.87/−10.10	57.99/−21.28	−5.92/65.52	11.88/55.30	21.66/54.88
T12	43.34/−27.35	25.38/−24.71	53.87/−16.47	−9.68/47.64	14.41/55.82	28.25/65.25

**Table 2:** Independent descriptive CIELAB extrema within each matrix. The table reports the scenario with the lowest observed L* and the scenario with the highest observed C*ab as separate dimensions; it does not apply a composite score or identify a preferred treatment. °Hue is reported for the highest chroma observation to complete the CIELAB description. Values are not dry-matter-normalised, and optical attenuation may be influenced by turbidity or scattering. Censored spectra are flagged and are not used quantitatively.

Descriptive Interpretation	Optical Limitation	Highest Observed C*ab/°Hue	Lowest Observed L*	Matrix
Independent observed CIELAB extrema; no composite ranking	No censored points in these two records	T4; C*ab = 55.92; °Hue = 169.97°	T12; L* = 15.46	Red cabbage
Descriptive observation from one preparation	Turbidity and scattering were not assessed	T12; C*ab = 35.42; °Hue = 315.77°	T12; L* = 71.18	Iris flower
Qualitative only; no within-matrix ranking	Preprocessing was not traceable; T2 contains 3 censored points	T10; C*ab = 75.37; °Hue = 346.00°	T2; L* = 34.06	Dragon fruit
Descriptive CIELAB extrema; spectral index not used	T8 contains 1 censored wavelength point	T8; C*ab = 126.18; °Hue = 86.11°	T4; L* = 75.17	Orange peel
Descriptive observation; darkness may include scattering	T8 contains 3 censored points; turbidity unmeasured	T7; C*ab = 77.04; °Hue = 70.13°	T8; L* = 25.32	Spent coffee grounds
Descriptive observation; no extraction-efficiency claim	Several scenarios contain censored points	T2; C*ab = 93.13; °Hue = 54.62°	T4; L* = 3.81	Pine bark

**Table 3:** Extraction treatments applied to each matrix. The rows are composite screening scenarios in which several variables may differ simultaneously; they are not factorial main-effect contrasts.

Treatment	Extraction Medium	Time and Temperature/Pretreatment	Combined Scenario Descriptor
T1	Water	45 min; prior freezing	Cell disruption by freezing
T2	Water	45 min; room temperature	Mild aqueous extraction
T3	Ethanol	10 min; 70 °C	Heated polar organic extraction
T4	Ethanol + 100 µL 30% KOH stock in 20.0 mL (0.50% *v*/*v* stock; stock basis not preserved)	10 min; 70 °C	KOH-stock added; final pH unmeasured
T5	Ethanol + 100 µL HCl 1 N in 20.0 mL (0.005 N nominal)	10 min; 70 °C	HCl-stock added; final pH unmeasured
T6	Ethanol/glacial acetic acid 80:20 *v*/*v* (20% *v*/*v* acetic acid)	10 min; 70 °C	Concentrated ethanol–acetic acid mixture
T7	Water	10 min; 100 °C	Boiling water extraction
T8	Working medium labelled “30% acetone” (basis not preserved)	10 min; 70 °C	Heated acetone-containing scenario; exact concentration unavailable
T9	Ultrasound in water	10 min; room temperature	Ultrasound-assisted aqueous extraction
T10	Ultrasound in water	10 min; 70 °C	Ultrasound plus heating
T11	Ultrasound in water	10 min; room temperature after freezing	Ultrasound after prior freezing
T12	Ultrasound in water	10 min; 70 °C after freezing	Ultrasound, heating and prior freezing

## Data Availability

All point-by-point data supporting the spectral analyses are provided with the article. Supplementary Data File S1 contains the complete measured transmittance dataset and revised apparent-optical-attenuation dataset for 72 plant/agro-food matrix–scenario combinations in the main analysis plus 12 *Pisolithus* comparator combinations retained only in the Supplementary Materials (360–740 nm at 10 nm intervals), together with separate CIELAB coordinates, censoring flags and qualified descriptive optical indices. No composite intensity score or treatment-ranking field is retained. Supplementary Figures S1–S7 provide the corresponding complete paired spectral profiles, and Supplementary Figure S8 provides additional qualitative laboratory photographs.

## Supplementary Materials

The following supporting information can be downloaded at: https://www.mdpi.com/article/10.3390/plants15172685/s1. Table S1. Extraction-treatment key. The nominal 0.2 g inputs were weighed as received; moisture content was not determined, so no dry-matter-normalised extraction yield or cross-matrix efficiency comparison is provided. For dragon fruit, the exact treatments filtered or diluted, filter characteristics, dilution factors and concentration back-corrections were not preserved. These data are therefore retained for qualitative optical transparency but are not used for treatment ranking. The previous equal-weight low-L*/high-C*ab intensity score and all selection-status labels have been removed, and the CIELAB coordinates are reported separately. No extraction yield, pigment concentration, recovery percentage, mass balance, purity or application performance was determined; the supplementary data document the measured optical responses and do not quantify colorant recovery or identify pigment molecules. Figure S1. Complete visible optical profiles of red cabbage extracts for treatments T1–T12. (A) Original measured transmittance (%T), 360–740 nm. (B) Apparent optical attenuation derived as Aapp(λ) = −log10[%T(λ)/100] only where measured %T > 0.01. Values at or below 0.01%T are censored, omitted from the attenuation curves and marked with ×; no artificial Aapp = 4.00 ceiling is imposed. Line colour and style jointly identify treatments. No smoothing, composite score or treatment ranking is applied. The attenuation may contain contributions from absorption and scattering because turbidity was not independently measured and no validation against a conventional UV–visible spectrophotometer was performed. Figure S2. Complete visible optical profiles of Iris flower extracts for treatments T1–T12. (A) Original measured transmittance (%T), 360–740 nm. (B) Apparent optical attenuation derived as Aapp(λ) = −log10[%T(λ)/100] only where measured %T > 0.01. Values at or below 0.01%T are censored, omitted from the attenuation curves and marked with ×; no artificial Aapp = 4.00 ceiling is imposed. Line colour and style jointly identify treatments. No smoothing, composite score or treatment ranking is applied. The attenuation may contain contributions from absorption and scattering because turbidity was not independently measured and no validation against a conventional UV–visible spectrophotometer was performed. Figure S3. Complete visible optical profiles of dragon fruit extracts for treatments T1–T12. (A) Original measured transmittance (%T), 360–740 nm. (B) Apparent optical attenuation derived as Aapp(λ) = −log10[%T(λ)/100] only where measured %T > 0.01. Values at or below 0.01%T are censored, omitted from the attenuation curves and marked with ×; no artificial Aapp = 4.00 ceiling is imposed. Line colour and style jointly identify treatments. No smoothing, composite score or treatment ranking is applied. The attenuation may contain contributions from absorption and scattering because turbidity was not independently measured and no validation against a conventional UV–visible spectrophotometer was performed. Treatment-specific filtration, filter characteristics, dilution factors and concentration back-corrections were not preserved; the profiles are qualitative and are not used for within-matrix quantitative comparison. Figure S4. Complete visible optical profiles of orange peel extracts for treatments T1–T12. (A) Original measured transmittance (%T), 360–740 nm. (B) Apparent optical attenuation derived as Aapp(λ) = −log10[%T(λ)/100] only where measured %T > 0.01. Values at or below 0.01%T are censored, omitted from the attenuation curves and marked with ×; no artificial Aapp = 4.00 ceiling is imposed. Line colour and style jointly identify treatments. No smoothing, composite score or treatment ranking is applied. The attenuation may contain contributions from absorption and scattering because turbidity was not independently measured and no validation against a conventional UV–visible spectrophotometer was performed. Figure S5. Complete visible optical profiles of spent coffee grounds extracts for treatments T1–T12. (A) Original measured transmittance (%T), 360–740 nm. (B) Apparent optical attenuation derived as Aapp(λ) = −log10[%T(λ)/100] only where measured %T > 0.01. Values at or below 0.01%T are censored, omitted from the attenuation curves and marked with ×; no artificial Aapp = 4.00 ceiling is imposed. Line colour and style jointly identify treatments. No smoothing, composite score or treatment ranking is applied. The attenuation may contain contributions from absorption and scattering because turbidity was not independently measured and no validation against a conventional UV–visible spectrophotometer was performed. Figure S6. Complete visible optical profiles of pine bark extracts for treatments T1–T12. (A) Original measured transmittance (%T), 360–740 nm. (B) Apparent optical attenuation derived as Aapp(λ) = −log10[%T(λ)/100] only where measured %T > 0.01. Values at or below 0.01%T are censored, omitted from the attenuation curves and marked with ×; no artificial Aapp = 4.00 ceiling is imposed. Line colour and style jointly identify treatments. No smoothing, composite score or treatment ranking is applied. The attenuation may contain contributions from absorption and scattering because turbidity was not independently measured and no validation against a conventional UV–visible spectrophotometer was performed. Figure S7. Complete visible optical profiles of the Pisolithus benchmark extracts for treatments T1–T12. (A) Original measured transmittance (%T), 360–740 nm. (B) Apparent optical attenuation derived as Aapp(λ) = −log10[%T(λ)/100] only where measured %T > 0.01. Values at or below 0.01%T are censored, omitted from the attenuation curves and marked with ×; no artificial Aapp = 4.00 ceiling is imposed. Line colour and style jointly identify treatments. No smoothing, composite score or treatment ranking is applied. The attenuation may contain contributions from absorption and scattering because turbidity was not independently measured and no validation against a conventional UV–visible spectrophotometer was performed. This non-plant figure is retained solely as a supplementary comparator and is not included in the main plant-focused comparisons. Figure S8. Additional representative laboratory photographs of the evaluated matrices and extract sets. The images provide qualitative visual context for treatment-associated hue and intensity differences but were not used for quantitative image-based colour analysis. Labels identify the source matrix, and handwritten treatment numbers visible in the photographs correspond to treatments T1–T12 in Table S1. Data File S1. Supplementary_Data_File_S1.xlsx. The workbook contains the complete unchanged measured transmittance dataset comprising 3276 point-by-point values, 3241 calculable apparent-optical-attenuation values, 35 censored points, the extraction-treatment key, separate CIELAB coordinates, censoring and interpretation flags, qualified descriptive optical indices and a coverage summary. The previous intensity-score and selection-status fields have been removed. The workbook contains 72 plant/agro-food matrix–scenario combinations used in the main analysis and 12 Pisolithus combinations retained solely as the supplementary non-plant comparison.
