# Supplementary material for: Visible Spectral, CIELAB and Putative Chromophore-Class Screening of Plant and Agro-Food Residue Extracts
Source: Plants (Basel). 2026 Sep 1;15(17):2685. doi: 10.3390/plants15172685 (PMC13568250; doi:10.3390/plants15172685)
Supplement: Supplementary file 1 [file plants-15-02685-s001.zip › Supplementary Materials.pdf]

Supplementary Materials

Visible Spectral, CIELAB and Putative Chromophore-Class Screening of Plant and Agro-Food Residue Extracts

Joan Masanet Martí, Antonio Belda-Antolí, Daniel López Rodríguez, Jorge Jordán-Núñez, Álvaro-Francisco Morote Seguido and Bàrbara Micó-Vicent

Spectroscopic coverage. Figures S1–S7 provide the complete measured visible-transmittance and revised apparent-optical-attenuation profiles for all 84 matrix–treatment combinations (7 matrices × 12 treatments). Each spectrum contains 39 wavelength points from 360 to 740 nm at 10 nm intervals. No spectral smoothing was applied. Aapp(λ) = −log10[%T(λ)/100] is reported only when measured %T > 0.01. Thirty-five points across nine matrix–treatment combinations were at or below this operational floor; those points are treated as censored, omitted from Aapp curves and marked with ×. No artificial Aapp ceiling of 4.00 is used in the revised figures or workbook. The unchanged point-by-point transmittance dataset and the qualified attenuation dataset are supplied as Supplementary Data File S1.

Scope note. Figures S1–S6 correspond to the six plant/agro-food matrices analysed in the main manuscript. Figure S7 and the Pisolithus rows in Supplementary Data File S1 are retained solely as a supplementary non-plant comparison and are not included in the main-text comparative summaries, figures, tables or conclusions. No standardized clarification, turbidity measurement, sedimentation assessment or validation against a conventional UV–visible spectrophotometer was available. The Aapp profiles therefore represent apparent optical attenuation that may combine absorption and scattering and are not used for quantitative treatment ranking.

Methodological metadata and transformation-control note. The nominal input was approximately 0.2 g per treatment, but exact preparation-specific masses were not retained. Particle-size specifications, freezing temperature/duration, ultrasonic-bath model/frequency/power, extraction-vessel geometry/headspace, agitation during passive/heated treatments, post-extraction storage, light/oxygen protection and time-to-measurement were not systematically documented. No matched dark, oxygen-excluded, time-zero or time-course controls and no chromatographic mass balance were obtained. The spectra therefore represent net endpoint optical attenuation that may include release, scattering, transformation and degradation; they do not measure extraction efficiency or demonstrate a chemical mechanism.

Supplementary Table S1. Extraction-treatment key The nominal 0.2 g inputs were weighed as received; moisture content was not determined, so no dry-matter-normalised extraction yield or cross-matrix efficiency comparison is provided. For dragon fruit, the exact treatments filtered/diluted, filter characteristics, dilution factors and back-corrections were not preserved; these data are retained for qualitative optical transparency but are not used for treatment ranking. The previous equal-weight low-L*/high-C*ab intensity score and all selection-status labels have been removed; CIELAB coordinates are reported separately. No extraction yield, pigment concentration, recovery percentage, mass balance, purity or application performance was determined; the supplementary data document measured optical responses and do not quantify colorant recovery or identify pigment molecules.

Treatment	Extraction medium	Time	Temperature / pretreatment	Key variable
T1	Water	45 min	Prior freezing	Cell disruption by freezing
T2	Water	45 min	Room temperature	Mild aqueous extraction
T3	Ethanol	10 min	70 °C	Heated polar organic extraction
T4	Ethanol + 100 µL 30% KOH stock in 20.0 mL (0.50% v/v stock; basis not preserved)	10 min	70 °C	KOH-stock added; final pH unmeasured
T5	Ethanol + 100 µL HCl 1 N in 20.0 mL (0.005 N nominal)	10 min	70 °C	HCl-stock added; final pH unmeasured
T6	Ethanol/glacial acetic acid 80:20 v/v (20% v/v	10 min	70 °C	Concentrated ethanol–acetic acid mixture

	acetic acid)			
T7	Water	10 min	100 °C	Boiling-water extraction
T8	Working medium labelled “30% acetone” (basis not preserved)	10 min	70 °C	Heated acetone-containing scenario; exact concentration unavailable
T9	Ultrasound in water	10 min	Room temperature	Ultrasound-assisted aqueous extraction
T10	Ultrasound in water	10 min	70 °C	Ultrasound plus heating
T11	Ultrasound in water	10 min	Room temperature after freezing	Ultrasound after prior freezing
T12	Ultrasound in water	10 min	70 °C after freezing	Ultrasound, heating and prior freezing

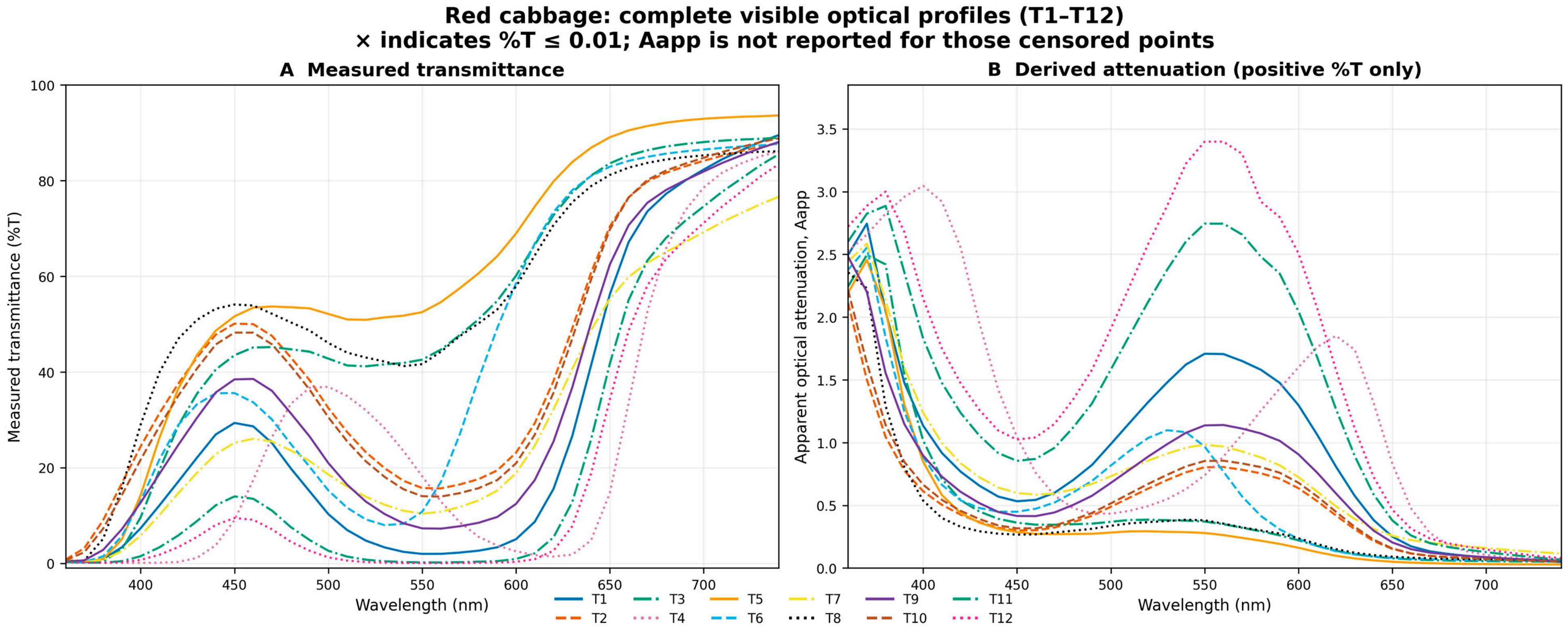

Figure S1. Complete visible optical profiles of Red cabbage extracts for treatments T1–T12. (A) Original measured transmittance (%T), 360–740 nm. (B) Apparent optical attenuation derived as Aapp(λ) = −log10[%T(λ)/100] only where measured %T > 0.01. Values at or below 0.01%T are censored, omitted from the attenuation curves and marked with ×; no Aapp = 4.00 ceiling is imposed. Line colour and style jointly identify treatments. No smoothing, composite score or treatment ranking is applied. The attenuation may contain contributions from absorption and scattering because turbidity was not independently measured and no conventional UV–visible validation was performed.

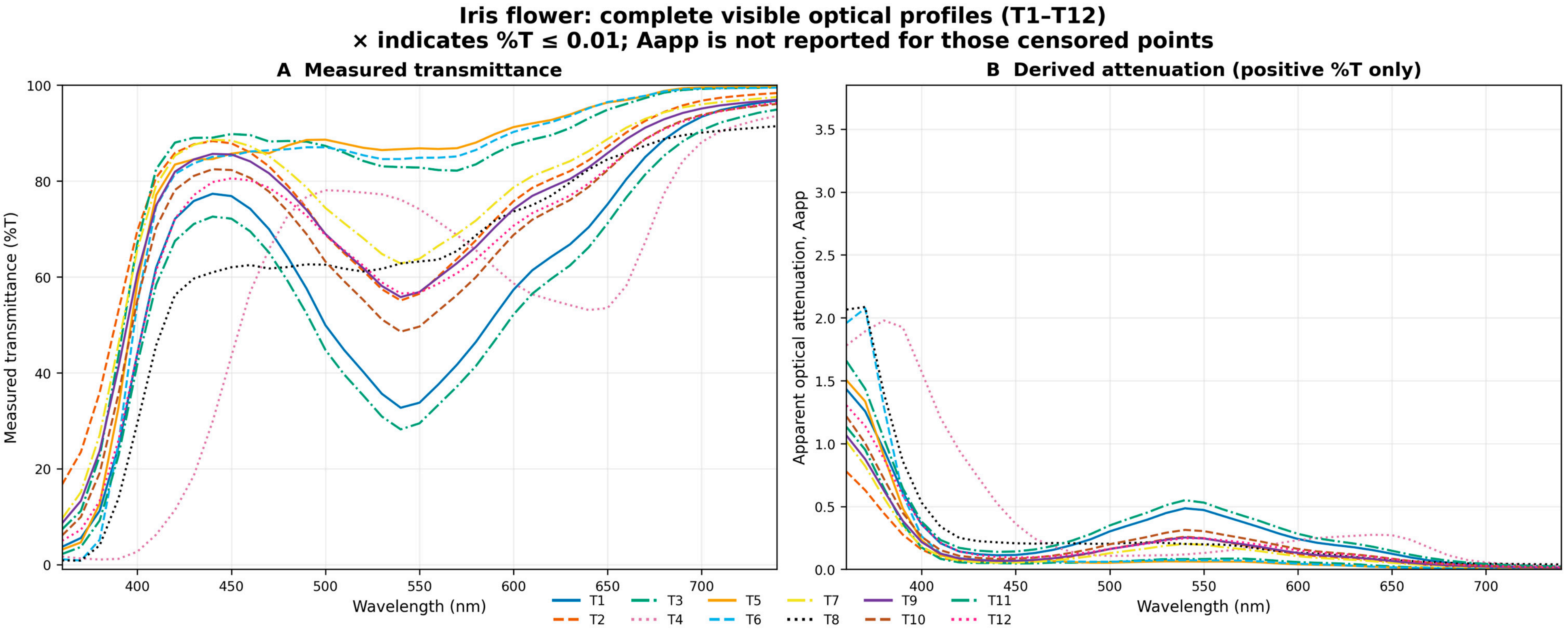

Figure S2. Complete visible optical profiles of Iris flower extracts for treatments T1–T12. (A) Original measured transmittance (%T), 360–740 nm. (B) Apparent optical attenuation derived as Aapp(λ) = −log10[%T(λ)/100] only where measured %T > 0.01. Values at or below 0.01%T are censored, omitted from the attenuation curves and marked with ×; no Aapp = 4.00 ceiling is imposed. Line colour and style jointly identify treatments. No smoothing, composite score or treatment ranking is applied. The attenuation may contain contributions from absorption and scattering because turbidity was not independently measured and no conventional UV–visible validation was performed.

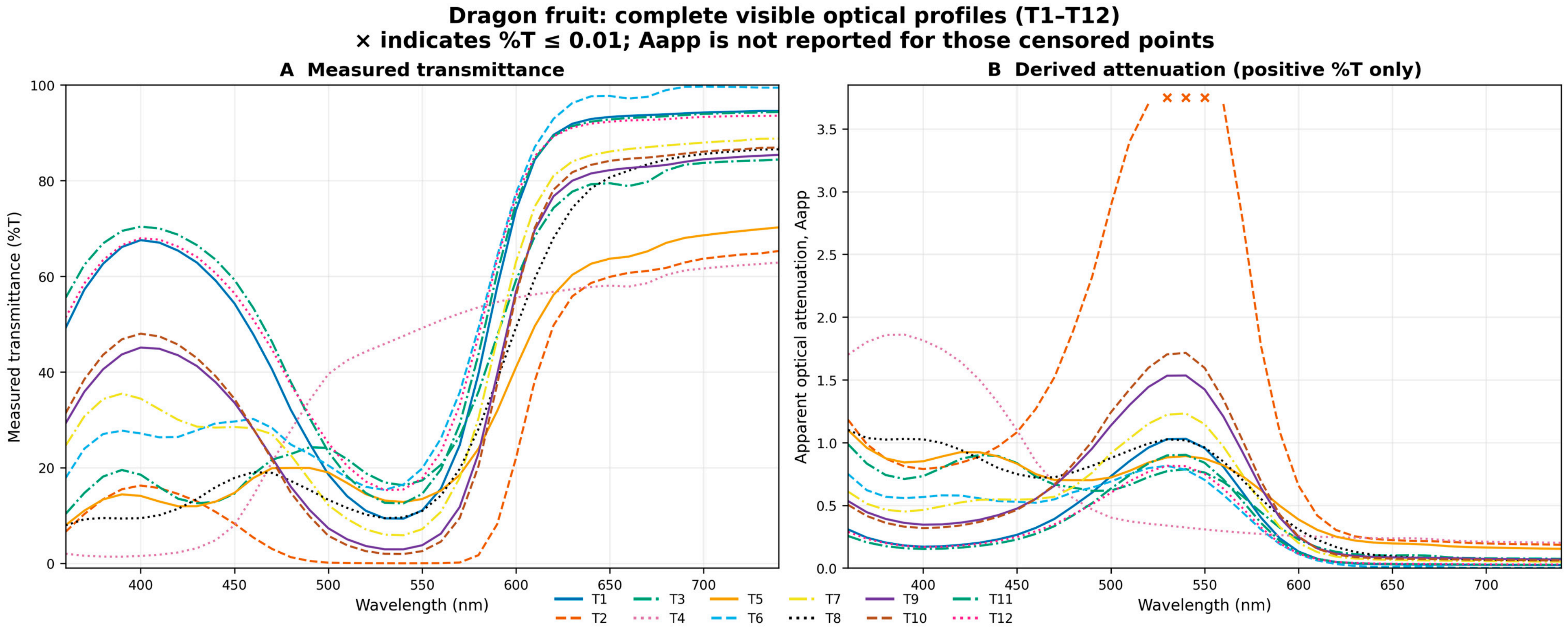

Figure S3. Complete visible optical profiles of Dragon fruit extracts for treatments T1–T12. (A) Original measured transmittance (%T), 360–740 nm. (B) Apparent optical attenuation derived as Aapp(λ) = −log10[%T(λ)/100] only where measured %T > 0.01. Values at or below 0.01%T are censored, omitted from the attenuation curves and marked with ×; no Aapp = 4.00 ceiling is imposed. Line colour and style jointly identify treatments. No smoothing, composite score or treatment ranking is applied. The attenuation may contain contributions from absorption and scattering because turbidity was not independently measured and no conventional UV–visible validation was performed. Treatment-specific filtration, filter characteristics, dilution factors and concentration back-corrections were not preserved; the profiles are qualitative and are not used for within-matrix quantitative comparison.

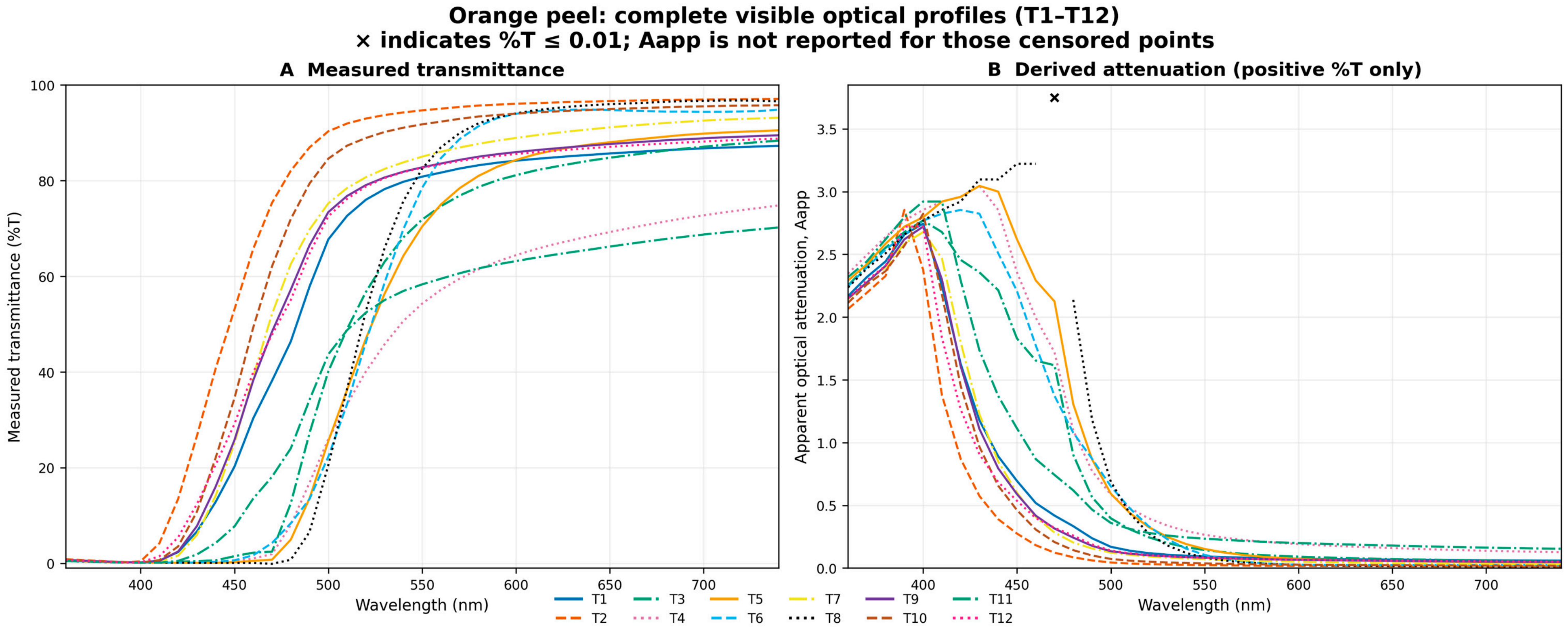

Figure S4. Complete visible optical profiles of Orange peel extracts for treatments T1–T12. (A) Original measured transmittance (%T), 360–740 nm. (B) Apparent optical attenuation derived as Aapp(λ) = −log10[%T(λ)/100] only where measured %T > 0.01. Values at or below 0.01%T are censored, omitted from the attenuation curves and marked with ×; no Aapp = 4.00 ceiling is imposed. Line colour and style jointly identify treatments. No smoothing, composite score or treatment ranking is applied. The attenuation may contain contributions from absorption and scattering because turbidity was not independently measured and no conventional UV–visible validation was performed.

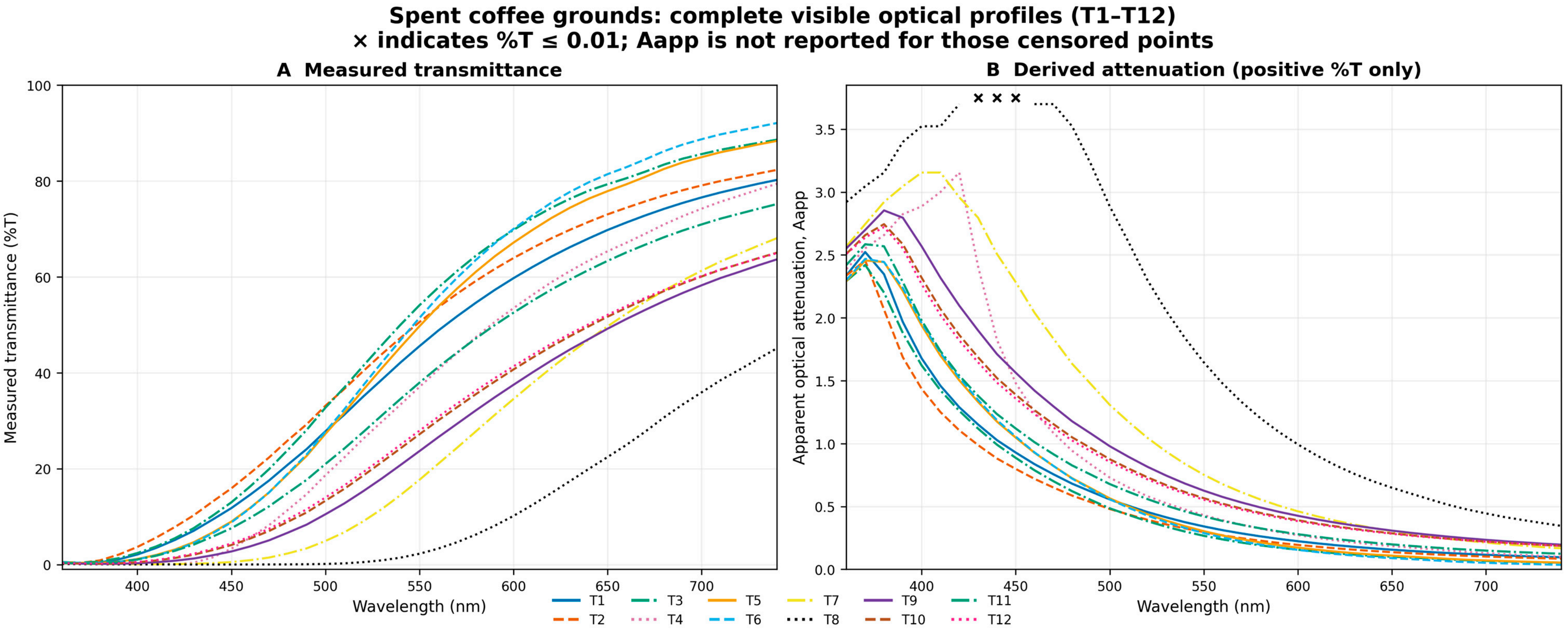

Figure S5. Complete visible optical profiles of Spent coffee grounds extracts for treatments T1–T12. (A) Original measured transmittance (%T), 360–740 nm. (B) Apparent optical attenuation derived as Aapp(λ) = −log10[%T(λ)/100] only where measured %T > 0.01. Values at or below 0.01%T are censored, omitted from the attenuation curves and marked with ×; no Aapp = 4.00 ceiling is imposed. Line colour and style jointly identify treatments. No smoothing, composite score or treatment ranking is applied. The attenuation may contain contributions from absorption and scattering because turbidity was not independently measured and no conventional UV–visible validation was performed.

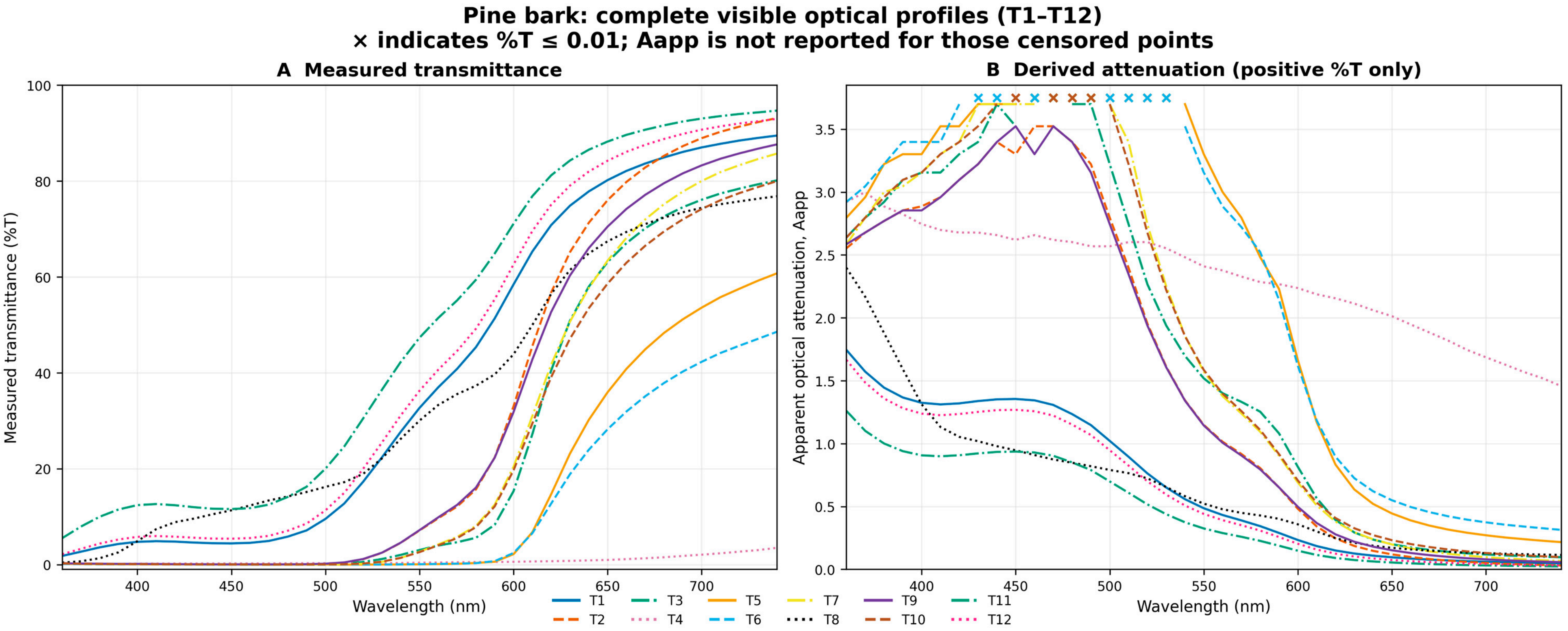

Figure S6. Complete visible optical profiles of Pine bark extracts for treatments T1–T12. (A) Original measured transmittance (%T), 360–740 nm. (B) Apparent optical attenuation derived as Aapp(λ) = −log10[%T(λ)/100] only where measured %T > 0.01. Values at or below 0.01%T are censored, omitted from the attenuation curves and marked with ×; no Aapp = 4.00 ceiling is imposed. Line colour and style jointly identify treatments. No smoothing, composite score or treatment ranking is applied. The attenuation may contain contributions from absorption and scattering because turbidity was not independently measured and no conventional UV–visible validation was performed.

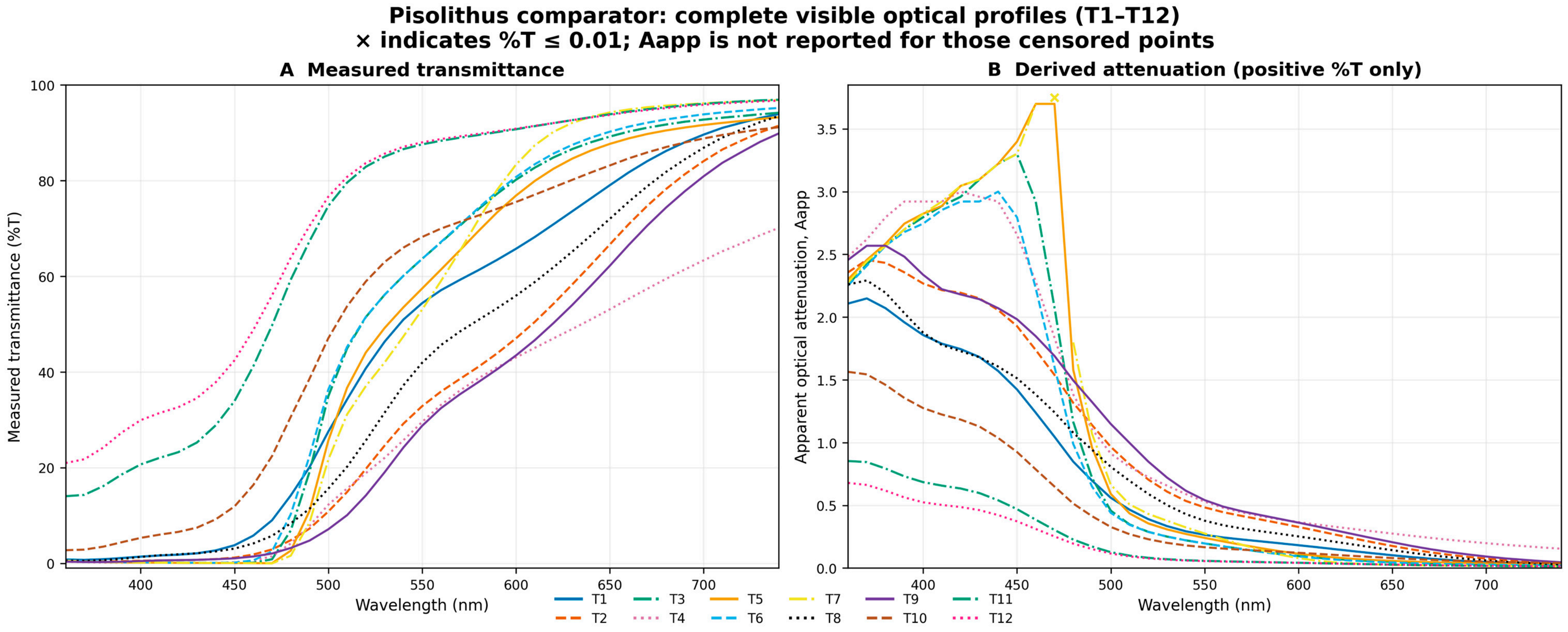

Figure S7. Complete visible optical profiles of Pisolithus benchmark extracts for treatments T1–T12. (A) Original measured transmittance (%T), 360–740 nm. (B) Apparent optical attenuation derived as Aapp(λ) = −log10[%T(λ)/100] only where measured %T > 0.01. Values at or below 0.01%T are censored, omitted from the attenuation curves and marked with ×; no Aapp = 4.00 ceiling is imposed. Line colour and style jointly identify treatments. No smoothing, composite score or treatment ranking is applied. The attenuation may contain contributions from absorption and scattering because turbidity was not independently measured and no conventional UV–visible validation was performed. This non-plant figure is retained only as a supplementary comparator.

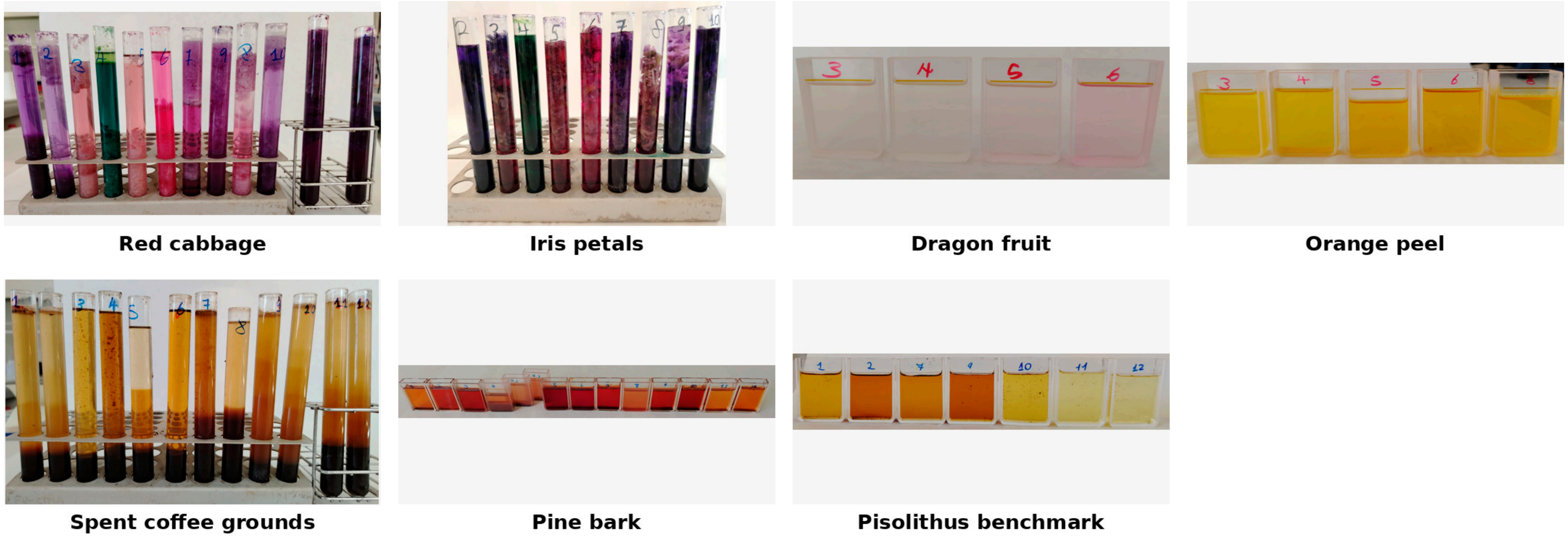

Figure S8. Additional representative laboratory photographs of the evaluated matrices and extract sets. The images provide qualitative visual context for treatment-dependent hue and intensity differences but were not used for quantitative image-based colour analysis. Labels identify the source matrix; handwritten treatment numbers visible in the photographs correspond to T1-T12 in Supplementary Table S1.

Supplementary Data File S1

Supplementary_Data_File_S1.xlsx contains the complete unchanged measured transmittance dataset (3276 values), 3241 calculable apparent-optical-attenuation values, 35 censored points, the treatment key, separate CIELAB coordinates, censoring/interpretation flags, qualified descriptive optical indices and a coverage summary. The previous intensity score and selection-status fields have been removed. The file contains 72 plant/agro-food matrix–scenario combinations used in the main analysis and 12 Pisolithus combinations retained only as the supplementary non-plant comparison.
